# Principled BCI Decoder Design and Parameter Selection Using a Feedback Control Model

**DOI:** 10.1038/s41598-019-44166-7

**Published:** 2019-06-20

**Authors:** Francis R. Willett, Daniel R. Young, Brian A. Murphy, William D. Memberg, Christine H. Blabe, Chethan Pandarinath, Sergey D. Stavisky, Paymon Rezaii, Jad Saab, Benjamin L. Walter, Jennifer A. Sweet, Jonathan P. Miller, Jaimie M. Henderson, Krishna V. Shenoy, John D. Simeral, Beata Jarosiewicz, Leigh R. Hochberg, Robert F. Kirsch, A. Bolu Ajiboye

**Affiliations:** 1 Department of Biomedical Engineering, Case Western Reserve University, Cleveland, Ohio USA; 2 Louis Stokes Cleveland Department of Veterans Affairs Medical Center, FES Center of Excellence, Rehab. R&D Service, Cleveland, Ohio USA; 3 Department of Neurosurgery, Stanford University, Stanford, California USA; 4 Department of Electrical Engineering, Stanford University, Stanford, California USA; 5 School of Engineering, Brown University, Providence, RI USA; 6 Center for Neurorestoration and Neurotechnology, Rehabilitation R&D Service, Department of Veterans Affairs Medical Center, Providence, RI USA; 7 Department of Neurology, University Hospitals Case Medical Center, Cleveland, Ohio, USA; 8 Department of Neurosurgery, University Hospitals Case Medical Center, Cleveland, Ohio, USA; 9 Stanford Neurosciences Institute, Stanford University, Stanford, 94305 California USA; 10 Department of Bioengineering, Stanford University, Stanford, California, 94305 USA; 11 Department of Neurobiology, Stanford University, Stanford, California, 94305 USA; 12 Howard Hughes Medical Institute, Stanford University, Stanford, California 94305 USA; 13 Neurosciences Program, Stanford University, Stanford, California 94305 USA; 14 Bio-X Program, Stanford University, Stanford, California 94305 USA; 15 Carney Institute for Brain Science, Brown University, Providence, Rhode Island USA; 16 Center for Neurotechnology and Neurorecovery, Department of Neurology, Massachusetts General Hospital, Boston, Massachusetts USA; 17 Department of Neurology, Harvard Medical School, Boston, Massachusetts, USA

**Keywords:** Brain-machine interface, Motor cortex

## Abstract

Decoders optimized offline to reconstruct intended movements from neural recordings sometimes fail to achieve optimal performance online when they are used in closed-loop as part of an intracortical brain-computer interface (iBCI). This is because typical decoder calibration routines do not model the emergent interactions between the decoder, the user, and the task parameters (e.g. target size). Here, we investigated the feasibility of simulating online performance to better guide decoder parameter selection and design. Three participants in the BrainGate2 pilot clinical trial controlled a computer cursor using a linear velocity decoder under different gain (speed scaling) and temporal smoothing parameters and acquired targets with different radii and distances. We show that a user-specific iBCI feedback control model can predict how performance changes under these different decoder and task parameters in held-out data. We also used the model to optimize a nonlinear speed scaling function for the decoder. When used online with two participants, it increased the dynamic range of decoded speeds and decreased the time taken to acquire targets (compared to an optimized standard decoder). These results suggest that it is feasible to simulate iBCI performance accurately enough to be useful for quantitative decoder optimization and design.

## Introduction

Intracortical brain-computer interfaces (iBCIs) can help to restore movement to people with severe paralysis by recording intact motor cortical signals and using them to guide the motion of an external device such as a robotic arm, a computer cursor, or muscle stimulators^1–7^. iBCIs are typically calibrated using statistical model fitting approaches that tune the decoder to predict, with minimum error, a set of intended movement variables given a set of neural features^1–3,8–16^. The calibration dataset could consist of neural recordings taken while the user observes, imagines or attempts to make a series of cued movements^2–4,17^. It could also be a dataset recorded during active iBCI control, in which case the user’s intended movement must be estimated at each time step (this approach is called “closed-loop calibration”^4,17–20^). Though it can yield high performing decoders (e.g.^3,6,19^), this traditional statistical model fitting approach to decoder calibration does not always maximize *online* performance. In other words, decoders that are optimal at predicting the intended movement variables in the calibration dataset do not necessarily maximize the ability of a person to actively *use* the decoder to complete a given task.

Several studies have now clearly shown a discrepancy between offline and online decoder performance. For example, one study demonstrated that medium bin widths (150 ms) optimize *offline* prediction performance for a Kalman filter while smaller bin widths (20 ms) optimize *online* performance, presumably because they enable the user to make feedback corrections more quickly^21^. Additionally, it has been shown that decoders with poor offline performance can have better online performance due to the user’s ability to adapt to certain kinds of consistent decoding errors^9,10^. Another study showed that unintended dynamics can result when decoders are calibrated to maximize offline performance and suggested that decoder dynamics should be optimized in a task-specific manner to achieve peak online performance^22^. Finally, we recently showed that many standard decoder calibration methods fail to optimize the decoder’s gain and smoothing parameters in a way that meaningfully accommodates the online task demands (e.g. by decreasing the gain and increasing smoothing for tasks that demand precision), even when closed-loop data is used for calibration^23^. The offline vs. online discrepancy demonstrated by the above studies is not entirely unexpected, given that traditional algorithms (e.g. the Kalman filter^19,22,24^) do not represent user behavior or task demands in their equations and do not model the feedback correction processes inherent to online control.

In our experience, to achieve peak performance certain decoder parameters like output gain (speed scaling) and temporal smoothing must be optimized through a process of trial and error where several different values are tested online. In this study, we asked: is there a way to predict which parameter settings will yield the best online performance without a trial and error process? Trial and error is time consuming and limits the number of parameter settings that can be tested. Cunningham *et al*. proposed using an “online prosthesis simulator” (OPS) to simulate the online/closed-loop dynamics resulting from a given parameter setting^21^. The OPS uses an able-bodied human volunteer to simulate the iBCI user. As the volunteer moves their arm, simulated neural activity is generated as a function of those movements and that simulated neural activity drives the decoder. Visual feedback of the decoder output is then given to the volunteer, closing the feedback control loop. Unlike offline prediction performance, this approach takes into account the feedback loop created by the user and the decoder, including how variability in the recorded neural activity creates movement errors and how the user adjusts their neural modulation to correct for those errors. It also automatically takes into account the specific requirements of the task that the decoder will be used to complete.

Here, we develop a similar simulation-based approach to predict online performance and validate it by comparing its predictions to held-out closed-loop iBCI data from three clinical trial participants. Our approach improves upon the OPS study by being able to run entirely on the computer (requiring no input from a human volunteer) and by enabling user-specific performance predictions. This expands the utility of the approach by enabling a rapid search across more parameters than would be possible with human volunteers. It also allows the simulation approach to be used in a clinical setting to customize the decoding parameters to suit a given iBCI user. Although several other studies have also successfully employed computer simulations of iBCI control to make qualitative insights (e.g.^22,25,26^), we are aware of no prior work that has demonstrated an ability to simulate iBCI control with the accuracy required for quantitative parameter selection and design.

Our simulator, which we call the PLM (piecewise-linear model), is based on a feedback control model of two-dimensional iBCI-commanded cursor movements that was developed in a previous study investigating how users control iBCIs^27^. In that study, we showed that the PLM outperformed other published models in its ability to simulate online performance under the same decoder and task parameters on which the model was fit. Here, we systematically assess, for the first time, how well a modified version of the model can *predict* ahead of time how online performance will change when decoder and task parameters are different from those under which the model was fit. Specifically, we test the model’s ability to predict how iBCI performance will change as a function of the gain and smoothing properties of a linear velocity decoder and as a function of target distance and radius. We evaluate the simulator by fitting its parameters with data collected under a single condition and then using it to predict how performance will change under various other held-out conditions. Finally, we demonstrate that a simulation-based approach has broader utility by using it to design a static nonlinear function that transforms the speeds decoded by a linear velocity decoder. We confirm that the nonlinear function improves online iBCI performance in a 2D and 4D cursor control task in two out of two participants and works well out-of-the-box without requiring any additional online parameter tuning.

## Results

### Standard calibration methods do not optimize decoder dynamics for online performance

**Figure 1:**
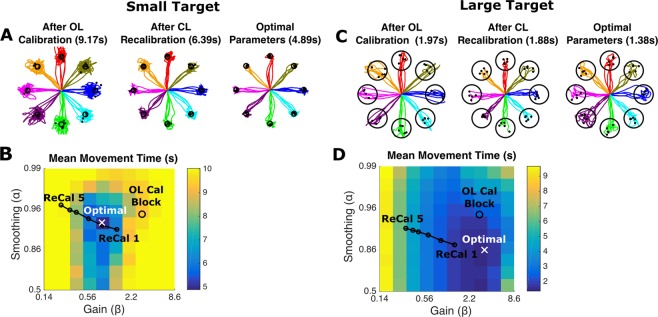
Standard calibration techniques do not always yield the optimal gain and smoothing properties for a velocity Kalman filter. Here, we define optimal as minimizing the mean movement time. **(A)** Simulated cursor movements using an initial decoder that was calibrated on open-loop (OL) data, using a decoder recalibrated with data from the first closed-loop (CL) block, and using a decoder with optimal gain and smoothing parameters. Average movement times are indicated in parentheses. **(B)** Average movement time as a function of gain and smoothing for this particular task and simulated user. Continued re-calibration of the decoder for 5 blocks (ReCal 1–5) does not cause the gain and smoothing values to converge to the optimal setting. **(C**,**D)** Same plots as in A and B except with a larger target radius; in this case, higher gain and lower smoothing values are optimal.

Before assessing the PLM’s ability to predict online performance under different decoder parameters, we first motivate the problem further with a simulated example of how a typical decoder calibration routine can fail to find the best gain and temporal smoothing parameters for online performance (Fig. 1). In a typical routine (e.g.^3,4,6,7^), an initial decoder is first calibrated on open-loop data where the user attempts to make a series of cursor movements shown on the screen. The decoder is then re-calibrated using closed-loop data recorded while the user makes movements with the initial decoder. We simulated this process using a simulated iBCI user with linearly tuned neural features that encoded a simple feedback control policy:

$${f}_{t}=E({g}_{t}-{p}_{t})+{\varepsilon }_{t}$$

where *f*_*t*_ is an N × 1 vector of neural features, *E* is an N × 2 matrix of tuning coefficients (with uniformly distributed preferred directions), *g*_*t*_ is the target position, *p*_*t*_ is the cursor position, and *ε*_*t*_ is neural variability (normally distributed). We calibrated velocity Kalman filters to predict the user’s intended velocity. The intended velocity was estimated by rotating the decoded velocity vectors in the calibration dataset towards the target and zeroing them when the cursor is on top of the target (following the ReFIT method described in^19^).

Open-loop calibration and closed-loop re-calibration yielded decoders with different gain and smoothing properties, but neither were optimal (i.e. did not minimize movement time when compared to the best parameter settings identified with an exhaustive parameter sweep). We performed the same simulation with both a small and a large target radius. For the small radius task, the standard training protocol yielded a gain that was too high with smoothing that was too low. This resulted in trajectories that orbited around the target instead of stopping. For the large radius task, the gain was too low and the smoothing was too high, resulting in trajectories that were slower than necessary. Continued closed-loop recalibration did not cause the decoder dynamics to converge towards optimal values; instead, the gain continued to decrease and the smoothing continued to increase without bound (shown as ReCal 1–5 in Fig. 1 and previously noted in^18^). To confirm that this result is not limited to the ReFIT calibration method, we ran the same simulation using the encoded control signal ${g}_{t}-{p}_{t}$ to calibrate the decoder and found similar results (Supplemental Fig. 1). These results are consistent with recent work that showed a similar inability to optimize gain and smoothing when comparing across different decoder calibration methods^23^. More details about the simulation parameters are provided in Section 1 of the Supplement. In sum, these results demonstrate that existing decoder calibration protocols are suboptimal, and motivate the need for a better way to set decoder gain and smoothing.

### Predicting how gain and smoothing affect online performance

**Figure 2:**
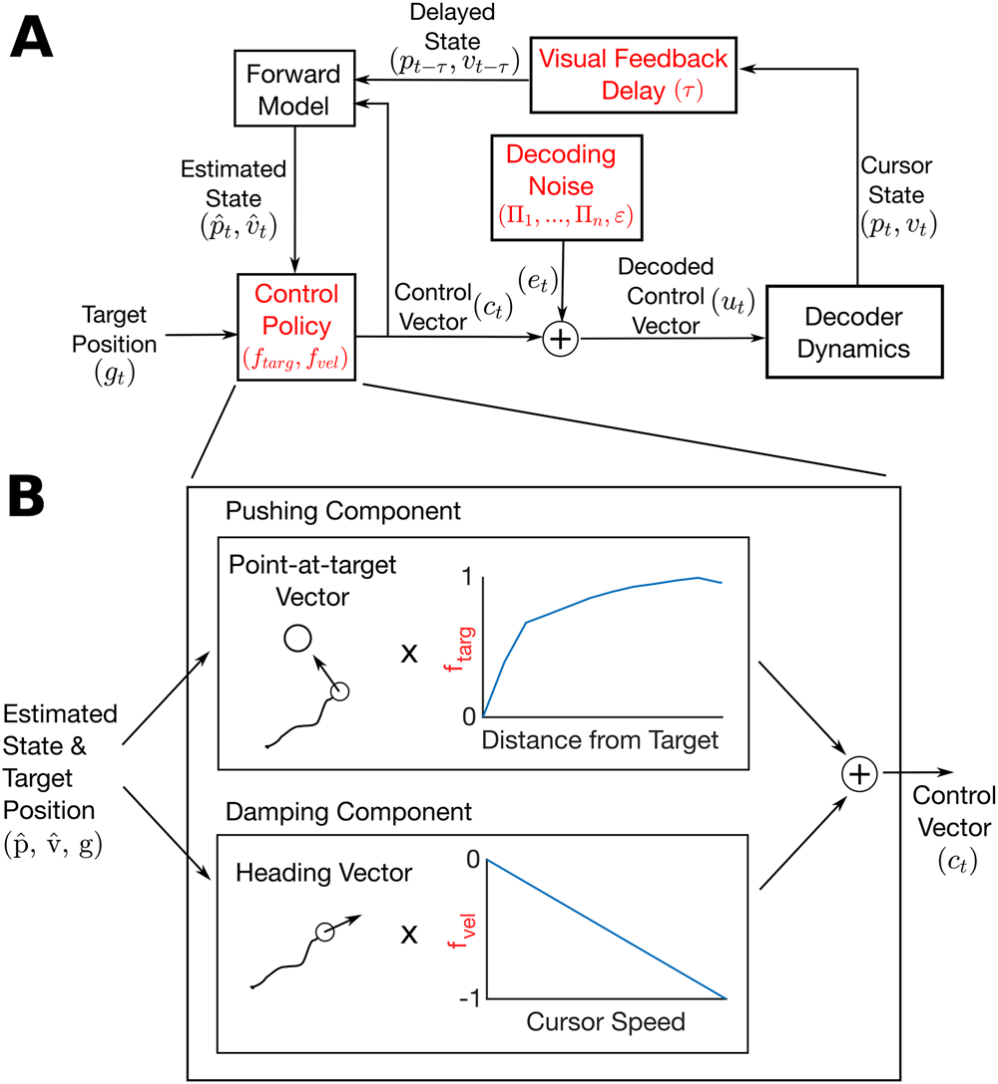
Illustration of the piecewise-linear feedback control model (PLM) used to simulate iBCI performance in this study. (**A**) Diagram of the feedback control model, with elements that are customized to each user shown in red. In the model, the simulated user receives delayed visual feedback of the cursor position and velocity. From the delayed feedback, the simulated user employs a forward model to estimate the current state of the cursor. The estimated cursor state and target position are then used to compute a control vector based on the user’s control policy. Finally, decoding noise, parameterized by an autoregressive model, is added to the control vector to simulate the decoder output. The decoder output is smoothed by first order smoothing dynamics and integrated to yield the cursor state. (**B**) Illustration of the control policy block. The user’s control vector is computed as the sum of a pushing component (a point-at-target vector weighted by the piecewise linear function f_targ_) and a damping component (a heading vector weighted by the piecewise linear function f_vel_). The pushing component causes the cursor to move towards the target and the damping component slows down the cursor as needed to avoid overshooting the target (note that f_vel_ is negative).

If the effect of gain and smoothing can be predicted ahead of time through simulation, then high-performing values can be found through a simulated sweep without an online trial and error process. Here, we assess the ability of the piecewise-linear model (PLM) to simulate and predict online performance under different gain and smoothing parameters. The PLM’s parameters capture the characteristics of both the decoder and the iBCI user and are illustrated in Fig. 2. The PLM is fit to an individual iBCI user from previously collected decoder output and cursor kinematics data, but it then requires no new neural or behavioral data to simulate movements even for different decoder parameters or tasks. At each time step of the simulation, the two-dimensional decoder output *u*_*t*_ is simulated using a model of how the user controls the cursor combined with a model of the decoding noise. Individual neural features are not simulated. More details are provided in the Methods.

To test the PLM’s ability to predict the effect of gain and smoothing on iBCI performance, we analyzed 15 sessions of data in which participants completed a target acquisition task with a linear velocity decoder under different gain and smoothing conditions. These sessions included a total of 134 blocks of data across three participants, each with a different gain and smoothing setting. Gain was controlled with a speed scaling parameter and smoothing was set with an exponential smoothing parameter. Each of these blocks was collected after the decoder was calibrated and fixed for the remainder of the session. Each block lasted between 4 to 5 minutes.

For each session, the PLM’s parameters were fit to that session by selecting a single block of data (with a single gain and smoothing setting) and fitting the parameters using that block of data. To predict how online performance would change for each of the other held-out blocks, we used the PLM to simulate 1000 cursor movements under each block’s gain and smoothing settings and measured the performance of those simulated movements. Note that fitting a new model for each session was needed to adapt the model to the amount of decoding noise present on that day, which could vary considerably from session to session depending on what neurons were recorded on the array(s).

We used the following performance metrics to compare the predicted performance to the actual performance: total movement time, translation time (the time taken for the cursor to initially touch the target), dial-in time (the time taken to acquire the target after the cursor initially touches it, minus the obligatory dwell-time), and path efficiency (straightness of the cursor movement, measured by the distance of an ideal straight-line movement towards the target divided by the distance the cursor actually traveled).

**Figure 3:**
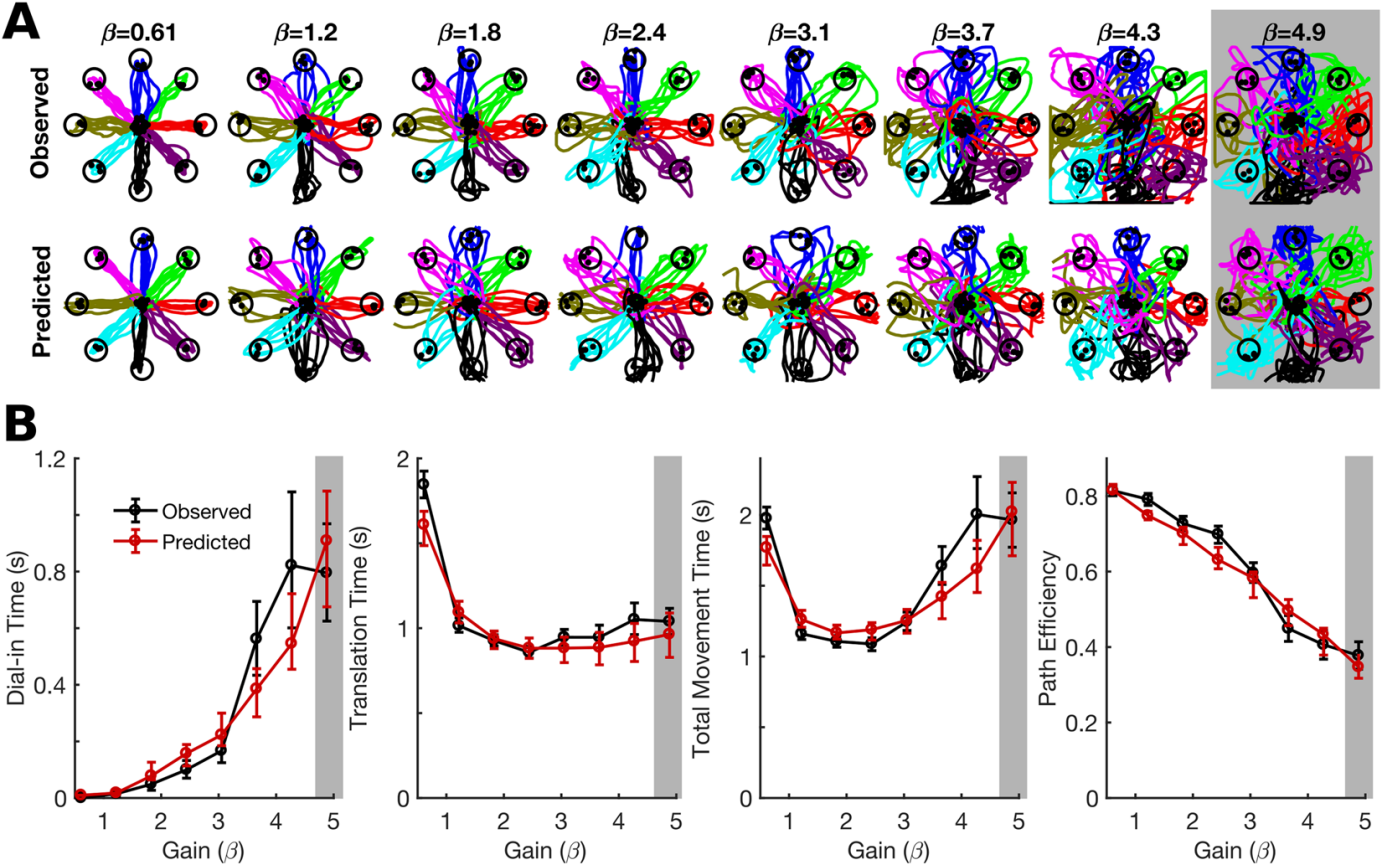
The PLM (piecewise-linear feedback control model) can predict the effect of gain on online performance. Results are shown for an example session with participant T6. **(A)** Observed cursor movements made during a single session under different gains (β, reported in units of target distances/second) are shown next to simulated trajectories predicted by the model. The model parameters were fit using data only from a single condition (β = 4.9, indicated in gray) and then used to simulate movements under different gain settings. **(B)** The data from (**A**) is quantified using four movement performance metrics (error bars represent 95% confidence intervals). Online performance is well predicted by the model (the red lines lie close to the black lines). Confidence intervals for model predictions were generated using bootstrap resampling (trials were resampled from each condition with replacement); the confidence intervals represent uncertainty in the predictions due to limited training data.

Figure 3 shows example data of cursor movements made under different gains. As gain is increased, translation time decreases (the cursor reaches the target faster) while dial-in time increases (it’s harder to acquire the target) and path efficiency decreases (movements are more indirect). The tradeoff between translation time and dial-in time causes the total movement time to be a U-shaped function of gain, with the optimal point at an intermediate gain setting. The PLM accurately predicts the effect of gain, including the tradeoff between translation time and dial-in time. Note that the model’s predictions are a close *quantitative* match to the observed performance (i.e. the model is doing more than just predicting the *qualitative* trends that result when varying a given parameter). This suggests that the model could be useful for predicting user-specific and task-specific parameters that will work out-of-the-box without the need for online customization.

**Figure 4:**
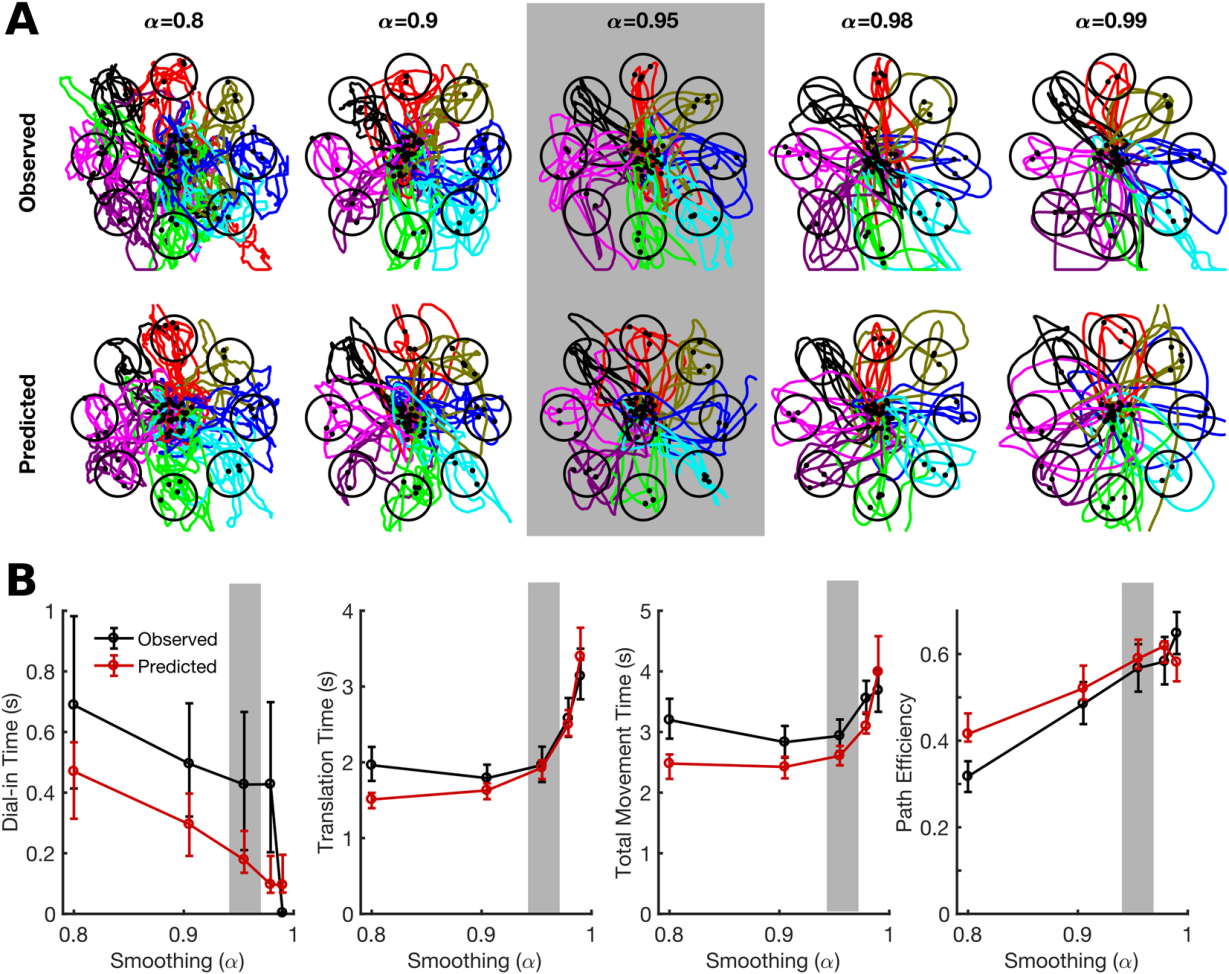
The PLM can predict the effect of temporal smoothing on online performance. Results are shown for an example session with participant T8. **(A)** Observed cursor movements made during a single session under different smoothing settings (α) are shown next to simulated trajectories predicted by the model. The model parameters were fit using data only from a single condition (α = 0.95, indicated in gray) and held fixed when simulating movements under different smoothing settings. **(B)** The data from (**A**) is quantified using four movement performance metrics (error bars represent 95% confidence intervals). Online performance is well predicted by the model (the red lines lie close to the black lines). Confidence intervals for model predictions were generated using bootstrap resampling (trials were resampled from each condition with replacement); the confidence intervals represent uncertainty in the predictions due to limited training data.

Figure 4 shows example data illustrating the effect of smoothing on online performance. Smoothing has the opposite effect of gain: as smoothing is increased, translation time increases (the cursor takes longer to accelerate) while dial-in time decreases and path efficiency increases (movements become straighter and stop more completely). The PLM is largely accurate at predicting how smoothing will affect online performance, though in this case it somewhat overestimates movement speed and straightness for the condition with the lowest smoothing.

**Figure 5:**
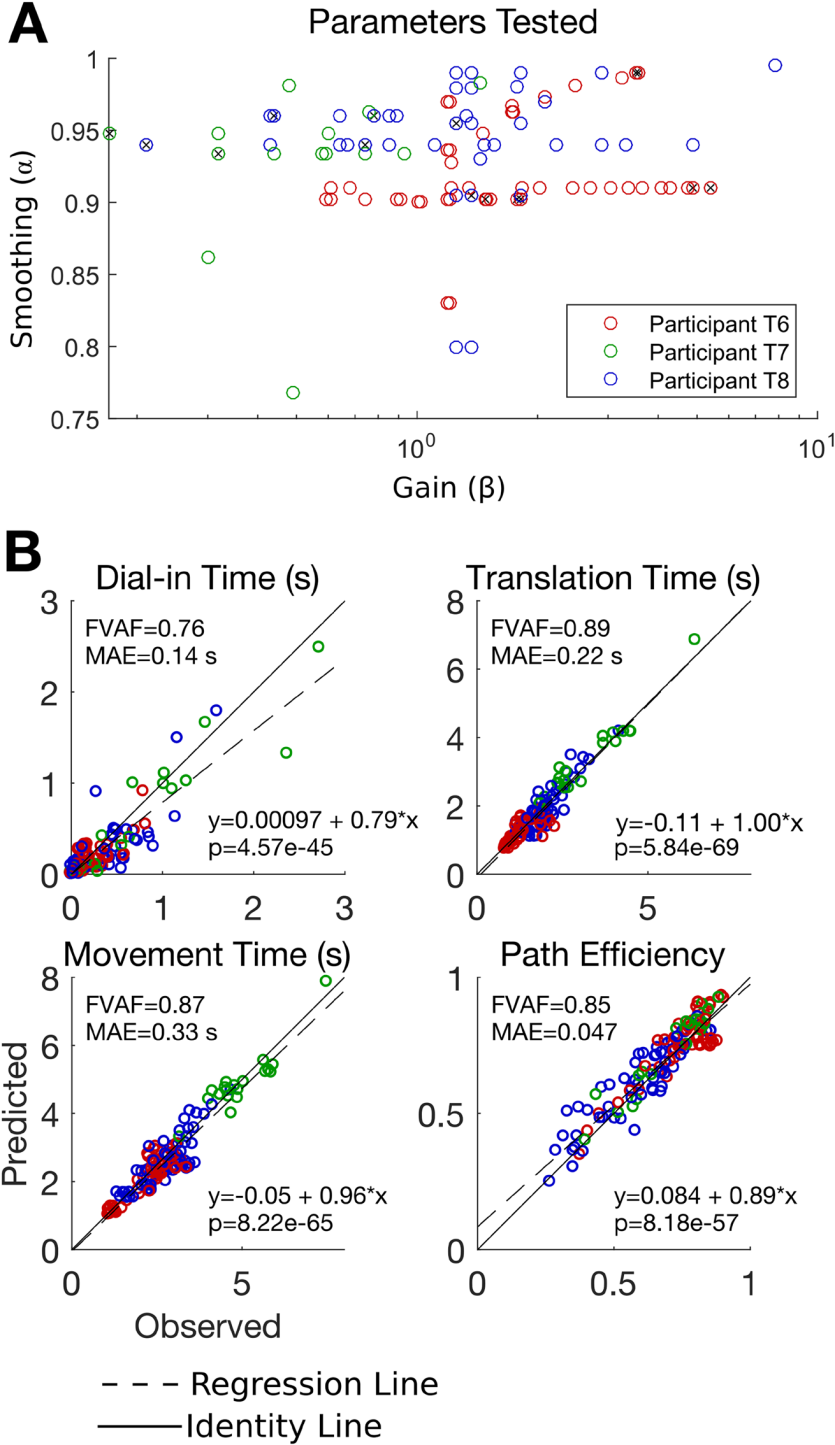
Assessing the ability of the PLM to predict online performance as a function of gain and smoothing across all 134 blocks included in the study. (**A**) The gain and smoothing settings imposed for each block are plotted as a circle. Blocks used to fit the model parameters are indicated with a black “x” inside the circle. (**B**) Observed vs. predicted online performance quantified using four movement performance metrics. Each circle represents the average performance for one block. Model predictions are a good quantitative match to the observed online performance (dots lie close to the solid unity line). In the top left corner of each panel, the fraction of variance accounted for by the model’s predictions (FVAF) is shown in addition to the mean absolute error of the predictions (MAE). To assess the model’s bias and statistical significance, a linear regression was performed for each panel that regressed the model’s predictions against the observed data. The regression coefficients are shown in the bottom right corner and indicate low bias (the slopes are near one and the intercepts are near zero). The regression line is plotted as a dashed black line and the unity line as a solid black line for comparison. Finally, the p-value for the slope coefficient is reported; the slope coefficients are statistically significant for all performance metrics (p < 1e-45 for all metrics), indicating that the model has statistically significant predictive power.

Figure 5 summarizes the PLM’s prediction performance for all 134 blocks. Overall, the model has good quantitative accuracy across a wide range of gain and smoothing parameters and three different users (e.g. the fraction of variance accounted for by the model’s predictions is greater than 0.7 for each performance metric). We assessed the bias of the model by fitting the model predictions as a linear function of the observed data (Fig. 5B). The results indicate slopes near 1 and intercepts near zero suggesting low bias. Very low p-values indicate that the model has statistically significant predictive power across all metrics (Fig. 5B).

Supplemental Section 5 shows the same data but for each session separately, confirming that the PLM can accurately predict within-session variance (due solely to gain and smoothing) in addition to explaining across-session variance (due partly to differences in participants and day to day changes in decoding noise^28^). We also confirmed that good performance across all participants could still be obtained regardless of which particular blocks for each session were used to fit the PLM. To do so, we compared model performance when the PLM was fit on the lowest gain block of each session, the highest gain block, and the median gain block. The average model FVAF and MAE did not vary appreciably (Supplemental Section 3).

### Predicting how task parameters affect online performance

Here, we test whether the PLM can predict how online performance will be affected when task parameters change (e.g. when the target distance or radius changes). This is important for two reasons: (1) the model should be able to predict optimal decoder parameters under a large variety of task settings (e.g. target distances and sizes), and (2) predicting how performance changes as a function of task parameters may enable principled optimization of the task (e.g. the sizes and placement of buttons on a virtual keyboard), complementing existing design approaches to user interfaces^29^.

**Figure 6:**
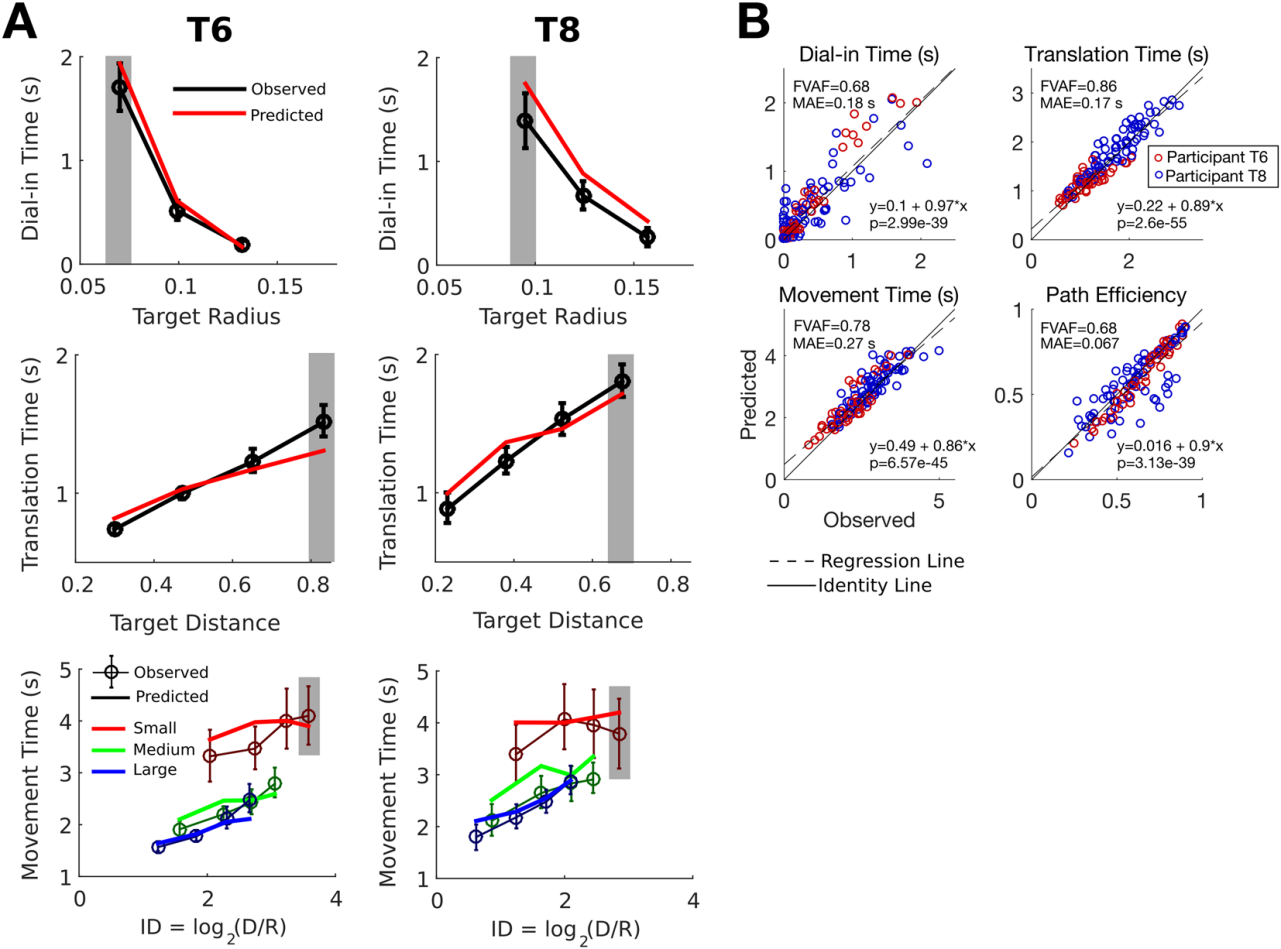
The PLM can predict online performance as a function of the task (target distance and target radius). (**A**) The model was fit only on movements to distant targets with small radii (indicated in gray). We illustrate observed vs. predicted performance on the random target task for an example session with T6 (left column) and T8 (right column). The model can predict how dial-in time increases as the radius becomes smaller (top row), how translation time increases as the target distance becomes greater (middle row), and how the total movement time is affected by both target distance and radius. In the bottom row, a separate index of difficulty (ID) vs. movement time line is drawn for each of the three target radii tested. The data show a departure from Fitts’ law that is predicted by the model (the departure is shown by the fact that ID does not fully predict movement time since the ID vs. movement time lines for each target radius do not lie on top of each other). (**B**) Observed vs. predicted online performance quantified using four movement performance metrics (same as in Fig. 5B). Each circle represents the average performance for one target distance and radius pairing. In the top left corner of each panel, the fraction of variance accounted for by the model’s predictions (FVAF) and the mean absolute error of the predictions (MAE) are shown. To assess the model’s bias and statistical significance, a linear regression was performed for each panel that regressed the model’s predictions against the observed data. The regression coefficients are shown in the bottom right corner and indicate low bias (the slopes are near one and the intercepts are near zero). The regression line is plotted as a dashed black line and the unity line as a solid black line for comparison. Finally, the p-value for the slope coefficient is reported.

We used 9 datasets where participants completed the random target task to test the PLM’s ability to predict how target distance and radius affect performance. For each dataset, we fit the model’s parameters to that dataset using only the movements made to targets placed far away from the cursor (with a distance in the top 25% of all tested distances) and with small radii (the smallest of three radii tested on each day). This set of movements constituted four minutes of data on average. We then used the fitted parameters to predict the online performance of movements made to all other targets (simulating 200 movements for each target distance and radius). Figure 6A shows example predictions for two of the datasets that demonstrate the accuracy of the PLM. Of note, the PLM correctly predicts the departures from Fitts’ law that are described in^30^ (the movement time vs. index of difficulty lines for each radius do not lie on top of each other), giving more confidence that the model describes iBCI movements well enough to extrapolate non-trivial results when fit on data from only a single condition. Figure 6B summarizes the prediction performance across all datasets and radius/distance pairings (3 radii and 4 distance categories per dataset = 12 circles per dataset). The model’s predictive power is highly statistically significant (p < 1e-38 for all metrics) and has relatively low bias and low error. Supplemental Section 5 shows that the prediction performance is also accurate for all 9 datasets considered separately.

### Using the PLM to choose gain and smoothing parameters and to optimize a typing interface

**Figure 7:**
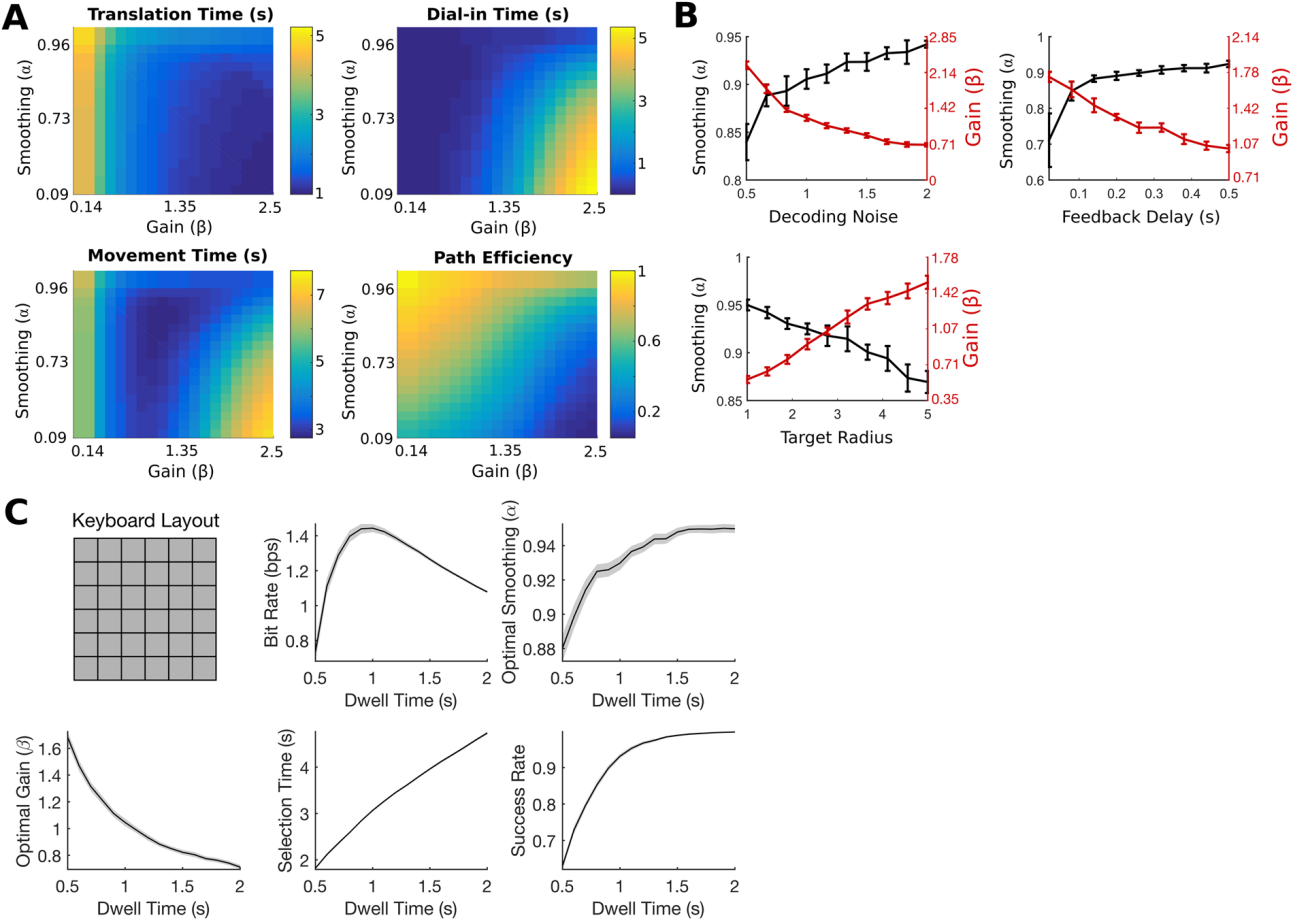
Example simulations illustrating how the PLM can be used to find optimal gain and smoothing parameters and to optimize a typing interface. (**A**) Cursor movements are simulated under different gain and smoothing values to generate two-dimensional performance surfaces that describe the average predicted performance as a function of gain and smoothing. A parameter pair can then be selected to optimize a chosen metric (e.g. average movement time) or any other desired criterion. The PLM parameters were fit on one example block of data collected with T8. (**B**) Illustration of how the optimal gain and smoothing parameters predicted by the PLM change as a function of different factors. Three factors are depicted: decoding noise variance, the user’s visual feedback delay, and target radius. The target distance was 14 units. Parameters were selected to optimize average movement time. Error bars represent 95% confidence intervals. The simulated optimization routine was repeated 10 times for each point. (**C**) Example of how the PLM can be used to optimize a typing interface (in this case a 36-key keyboard in a square layout). For each possible dwell time, a separate gain and smoothing optimization routine was performed. Bit rate, optimal gain and smoothing parameters, mean acquire time and success rate are reported for the gain and smoothing parameters that maximize bit rate at each dwell time. Shaded regions represent 95% confidence intervals. The simulated optimization routine was repeated 50 times for each point.

Once fit, the PLM can be used as a tool to choose decoder parameters that are likely to lead to higher performance online. In Fig. 7A we illustrate the process of how it can be used to find high-performing gain and smoothing parameters. Since movements can be simulated quickly, it is possible to simulate movements under a large number of settings to create performance surfaces describing how each performance metric is predicted to change as a function of gain and smoothing. Here, we simulated 250 movements for each of 400 pairs of gain and smoothing settings. The model parameters were fit on one example block of data collected with T8. The resulting performance surfaces reveal that, in general, path efficiency and dial-in time tradeoff with translation time. High gain and low smoothing is better for reaching the target more quickly (lower translation time), but worse for dwelling stably on the target (higher dial-in times and lower path efficiencies). Metrics that consider both the time taken to reach the target and the ease of acquiring the target (e.g. total movement time) have an optimal point that balances these trade-offs.

The PLM implicitly takes into account user-specific and task-specific aspects when predicting the optimal gain and smoothing parameters. Figure 7B illustrates this by showing how the gain and smoothing parameters chosen by the model vary as a function of decoding noise variance, feedback delay and target radius. Here, the optimal parameters were defined as those that were predicted to minimize the total movement time. In general, in more challenging settings (e.g. more decoding noise, long feedback delays, and small target radii), lower gain and higher smoothing values are better. In less demanding settings, higher gain and lower smoothing values are better. The PLM can take these factors into account to find the right parameter values for the specific situation. Note that, if optimal parameters are desired for a flexible task with varying target sizes and distances, these can be found by simulating trajectories towards targets with different radii and distances in whatever proportion the user would experience them and then averaging the performance metrics over these trajectories.

Next, we give an example of how the PLM can be used to help optimize a typing interface (Fig. 7C). It was recently shown that iBCIs can restore the ability to communicate at record speeds by combining 2D cursor control with an onscreen keyboard with a grid-like layout (shown in the top left of Fig. 7C)^18^. One method for key selection is to require the cursor to dwell on top of a key for a specified amount of time. How long should this dwell time be to maximize the information throughput? The dwell time presents a trade-off: shorter dwell times enable faster but less accurate selections, while longer dwell times enable slower but more accurate selections. In addition to the dwell time, the decoder parameters must also be optimized, and the optimal decoder parameters may change as a function of dwell time. This presents a difficult optimization problem that would be infeasible to fully explore without simulation. Moreover, the optimal dwell time and decoder parameters are likely to change from user to user since each user has a different amount of decoding noise. Thus, even if the time was spent to find good values for one user through trial and error, these would not necessarily be good values for other users.

We show how the PLM can be used to gain traction on this problem. First, we fit the PLM parameters to a block of data from participant T8. Then, for each possible dwell time (tested in steps of 100 ms) we performed a separate gain and smoothing optimization. For each gain and smoothing parameter pair (chosen on a 30 × 30 grid of possible pairs as in Fig. 7A), we measured the mean key selection time, success rate and overall information throughput (measured with the “achieved bit rate” metric that is a conservative lower bound on information throughput^29^). These performance metrics were computed by simulating a series of trajectories towards randomly chosen keys on the 36-key grid. Trajectories that accidentally selected the wrong key were counted as failures.

Figure 7C shows the results of this optimization, giving some insight into the factors at play in this optimization problem. The highest bit rate was achieved at a dwell time of 1 second where accuracy traded off optimally with speed; dwell times less than 1 second led to more accidental selections of nearby keys, while dwell times greater than 1 second caused selections to be unnecessarily slow. Note how the optimal gain and smoothing parameters changed with dwell time; longer dwell times benefited more from slower gains and more smoothing, which enabled the cursor to successfully dwell for a longer duration. Finally, it is interesting to note that the success rate is less than 1 at the optimal dwell time (success rate = 0.93 when dwell time = 1 s). This suggests that for optimal information throughput, the success rate should be lower than 1 (which is indicative of a regime where movements are fast enough to sometimes cause the occasional error). However, if a higher success rate is desired, the PLM can be used to select the dwell time needed to achieve the desired rate (for example, to achieve a success rate >0.99 a dwell time of 1.6 seconds is needed).

### Using the PLM to design and optimize a nonlinear speed transform

**Figure 8:**
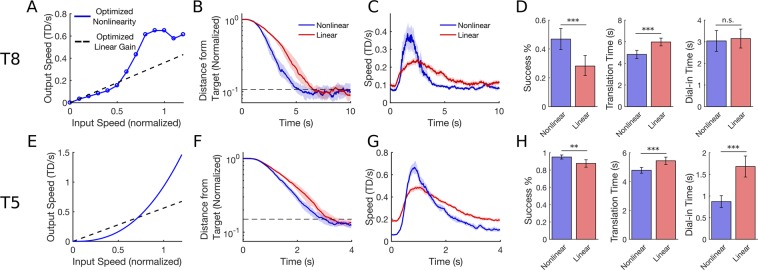
The PLM finds parameters for a nonlinear speed transform function that improve performance relative to an optimized linear decoder. Results from T8 are shown on the top row and results from T5 on the bottom. (**A**) The optimal nonlinearity found by the PLM keep low speeds low but scale up higher speeds significantly, enabling both precise stopping and quick movements to the target. (**B**) Median distance from the target as a function of time when using either the standard Kalman filter (red) or the Kalman filter with the nonlinear speed transform (blue). The added nonlinearity enables the user to reach the target more quickly while maintaining a similar stopping ability (the blue line crosses the target boundary first but levels off to the same steady-state distance as the red line). Shaded regions indicate 95% confidence intervals. Movements were pooled across two sessions. (**C**) Median cursor speed as a function of time. The nonlinearity improves performance by enabling faster speeds while traveling to the target without causing speeds to be faster when near the target. (**D**) Mean success %, translation time, and dial-in time with 95% confidence intervals. Three asterisks (***) indicate a significant difference with p < 0.001 and two asterisks (**) indicate p < 0.01 (as computed with a t-test for translation time and dial-in time and fisher’s exact test for success %). (**E–H**) Same as (**A–D**) but for participant T5.

Finally, we demonstrate that the PLM is also useful for designing new decoder improvements. Recent work has shown that linear decoders have a baseline floor of signal-independent decoding noise that makes it difficult for users to stop and make small corrective movements accurately^30^. To address this problem, we used the PLM to search for a nonlinear function that can improve stopping ability by transforming the speeds decoded by a standard linear velocity decoder. A nonlinearity might improve performance by scaling up higher speeds (allowing quick movements to the target) while still mapping a wide range of decoded speeds to lower values (to retain good stopping precision). We first tested this hypothesis with participant T8 (Fig. 8A–D) who performed a standard 2D cursor control task with or without the nonlinearity.

Figure 8A shows the resulting nonlinear function, found by iteratively searching for the parameters of a piecewise linear function that would optimize total movement time on a target acquisition task that demanded a relatively high amount of precision (the target radius + cursor radius was 1/8^th^ of the target distance and the dwell time was 4 seconds). Figure 8B,C show movements made by participant T8 with and without the nonlinearity. With the nonlinearity, T8 reaches the target more quickly than with a standard decoder but still retains the same stopping precision. Figure 8D confirms that there is a statically significant improvement in success rate and translation time with the nonlinearity.

We repeated these results in T5 with a few changes (Fig. 8E–H). First, we wanted to see if the results would generalize to higher dimensional tasks, including 3D movement and control of orientation (as would be required for controlling an arm). To test this, T5 completed a center-out-back 4D cursor control task where he was required to move a 3-dimensional bar to the location of a target bar and to rotate the bar to match the orientation of the target (using a single rotational degree of freedom). Second, we used a more constrained form of nonlinearity with a similar shape to that found in T8, but with the simpler form:

$${s}_{out}={{s}_{in}}^{p}$$

where *s*_*out*_ is the output speed, *s*_*in*_ is the input speed, and *p* is an exponentiation parameter which we optimized. The results are similar to that of T8, with the nonlinearity improving the dynamic range of speeds and improving stopping ability and decreasing the time taken to reach the target.

These results suggest that a simulation-based optimization approach may be useful in general whenever a parameter space is time consuming to search via trial and error alone. Because the simulator has good quantitative prediction accuracy (i.e. it is not just predicting qualitative trends), the parameters it chooses can work out-of-the-box without requiring additional hand tuning. Note that, although a straightforward idea like adding an exponential nonlinearity could have been conceived without the PLM, there would be no straightforward way to optimize it or accurately measure the performance improvement relative to an optimized linear decoder. In Supplemental Section 6, we discuss this further and illustrate that even a simple exponential nonlinearity creates a difficult joint optimization problem where decoder gain, smoothing and the exponent must be simultaneously optimized (because the optimal gain and smoothing values depend on the exponent).

### Model speed

For practical applications that use the PLM to tune decoder parameters for real-world use, it is important that the PLM parameters can be fit quickly and that movements are fast to simulate. The code we provide (https://github.com/fwillett/bciSim.git) can fit PLM parameters to 3 minutes of data in less than 30 seconds. Once fit, the PLM simulates cursor movements very quickly. Using an Intel i7-7920HQ processor (3.10 GHz) we were able to simulate 100 seconds of cursor movement (5,000 time steps) in only 3 milliseconds on average. This means that exhaustive grid searches over several parameters can be easily completed. For example, running a grid search over gain (20 values), smoothing (20 values), and the user’s velocity damping (10 values), we were able to sweep all 20 * 20 * 10 = 4,000 combinations with 250 movements per combination in 130 seconds on average (this parameter sweep is shown in Fig. 7A). Iterative searches that find local minimums in the space can reduce this time further; for example, a simple pattern search across these same parameters (using MATLAB’s “patternsearch”) finished in 3.4 seconds on average.

## Discussion

Online performance is not always optimized by standard decoder calibration methods (Fig. 1). Here, we used a feedback control model of iBCI cursor movements (called the “PLM” for piecewise linear model) to predict how online performance would change as a function of decoder parameters and task parameters. We showed that the PLM can predict how the gain and exponential smoothing properties of a linear velocity decoder affect online performance (Figs 3–5). It can also predict how target distance and target radius affects performance, giving confidence that the model can be used for a wide range of target parameters and may also be useful for optimizing user interfaces (Fig. 6). Finally, after validating the PLM we demonstrated how it can be used to predict optimal decoding parameters while automatically taking into account task-specific factors such as target radius and user-specific factors like decoding noise and feedback delay (Fig. 7). To show that a simulation-based optimization approach is viable for decoder parameters other than just gain and smoothing, we used the PLM to design a nonlinear function to transform the speeds decoded by a linear velocity decoder (Fig. 8). This function improved the user’s movement speed without sacrificing stopping precision.

### A new tool for predicting high-performing gain and smoothing parameters

The PLM can be used as a tool to automatically find gain and exponential smoothing parameters for a linear velocity decoder that are likely to have high online performance. We have shown that gain and smoothing have a significant effect on online performance, consistent with results from previous studies^10,22,30,31^. Thus, using the PLM to automatically customize them for a given task and user could yield significant performance benefits and eliminate the need for an online trial and error process. The model-based optimization process is quick (can be completed in <1 minute) and can be repeated daily, enabling automatic adaptation to changing neural signal quality over time. We hope that this new tool will improve the ability of paralyzed users to perform tasks on a computer (e.g. virtual typing^14^). The simulator, which is designed to be called from MATLAB, is publicly available on GitHub: https://github.com/fwillett/bciSim.git.

Beyond this practical application, there is also a research benefit for having an objective, automated way to tune the gain and smoothing properties of a linear velocity decoder to maximize performance for any given task and user. The PLM gives a straightforward way to help ensure that newly proposed decoders are compared to a high-performing linear decoder benchmark, so that any improvement shown is not simply due to how the new decoder alters gain or smoothing to outperform a poorly optimized decoder. Gain and smoothing have a large impact on performance^10,22,30,31^ and, unless carefully optimized or controlled, can bias the results of a study. For example, we recently compared different decoder calibration methods and showed that they yield essentially identical decoders that differ only in their gain and smoothing properties^23^. A naïve comparison between these methods showed large performance differences; however, when gain and smoothing properties were equalized, we saw virtually no performance difference between the methods.

### Nonlinear speed transform functions for improving linear velocity decoders

Reports have indicated that iBCI users can have difficulty stopping precisely when using standard linear decoders, including the steady-state velocity Kalman filter^30,32,33^. Recently, we showed that this was because of a floor of signal-independent decoding noise (i.e. noise that is independent of the user’s motor command) that persists even when the user attempts to slow down or stop^30^. To facilitate the acquisition of small targets when using standard linear decoders, the gain must be decreased to reduce the signal-independent noise enough so that the cursor can dwell comfortably within the small target. However, this then causes movements towards distant targets to become unnecessarily slow. A nonlinear transform applied to the output of the decoder may alleviate this problem somewhat by enabling both quick movements when traveling towards the target and slow movements when attempting to stop.

We demonstrated the utility of a simulation-based optimization approach by using the PLM to design such a transform. The resulting function scales up higher speeds (allowing quick movements to the target) while still mapping a wide range of decoded speeds to lower values (to retain the same or better stopping precision). Online results from participants T5 and T8 show that this approach is indeed one possible way to achieve a greater dynamic range of speed and precision than what a linear decoder can provide. Other solutions to this same problem have also been proposed, including attenuating the cursor speed when the cursor is changing directions more quickly^32^, using a nonlinear two state decoder that can switch between a postural decoder and a movement decoder^33^, and using a hidden Markov model to detect a stopping state^34^. Combining the nonlinear transform with these other improvements might yield greater gains in performance.

### Extending the model to more general decoding architectures

We showed that the PLM can predict closed-loop user behavior accurately when simulating a linear velocity decoder with exponential smoothing dynamics. Although this is a commonly used type of decoder that can achieve a high level of performance relative to its simplicity^1–4,6,13,15–17,20,35–37^, we anticipate that the field will ultimately move towards more general nonlinear decoders (e.g. neural networks^33,38–41^) that have a greater capacity to leverage patterns in the neural activity which lack a linear relationship to movement velocity. The same simulation-based design approach used here could be expanded to optimize more general kinds of decoders. Indeed, more complex decoders might stand to benefit even more from a simulation-based approach, since they have more free parameters that can be difficult to tune through trial and error alone.

To enable the PLM to optimize more general decoders, a more complete model of the user’s neural activity would have to be added. It would need to incorporate all aspects of the neural activity that the new decoder is designed to exploit. For example, it is known that certain dimensions of neural activity in motor cortex represent movement speed^41–43^ and movement timing^44^ in a way that is nonlinearly related to movement velocity. To simulate a neural network decoder that is capable of leveraging these aspects of the neural activity, neural tuning to movement speed and timing would first need to be added to the PLM model. One possible way to do this would be to simulate neural activity at each time step that is linearly tuned to the control vector computed by the PLM model (modeling standard “velocity” tuning), to the magnitude of the control vector (modeling “speed” tuning), and to a timing signal that unfolds as a function of time after the target appears. Once extended in this way, the PLM could be used to guide decoder design in the same way it was used here for linear decoders: by helping to optimize decoder parameters and explore new architectures. We hope that the proof-of-principle demonstrated here for linear decoders will inform a next generation model capable of enabling simulation-based design for the decoders of the future.

## Methods

### Study permissions and participants

This study includes data from four participants (identified as T5, T6, T7 and T8), who gave informed consent and were enrolled in the BrainGate2 Neural Interface System clinical trial (ClinicalTrials.gov Identifier: NCT00912041, registered June 3, 2009). This pilot clinical trial was approved under an Investigational Device Exemption (IDE) by the US Food and Drug Administration (Investigational Device Exemption #G090003). Permission was also granted by the Institutional Review Boards of University Hospitals (protocol #04-12-17), Stanford University (protocol #20804), Partners Healthcare/Massachusetts General Hospital (2011P001036), Providence VA Medical Center (2011-009), and Brown University (0809992560). All research was performed in accordance with relevant guidelines/regulations.

Participants were implanted with one (T6) or two (T5, T7, T8) 96 channel intracortical microelectrode arrays (Blackrock Microsystems, Salt Lake City, UT) in the hand area of dominant motor cortex (1.0-mm electrode length for T6, 1.5-mm length for T5, T7 and T8). All participants had chronic tetraplegia. T6 and T7 were diagnosed with Amyotrophic Lateral Sclerosis (ALS) and T5 and T8 were diagnosed with high level spinal cord injury. More details about each of the four study participants can be found in^6,27^.

### Dataset overview and relationship to previous studies

To validate the PLM, we leveraged datasets collected and reported in previous work^27,30^. We used 13 center-out-and-back datasets that were originally reported in^27^ to validate the model’s ability to predict performance as a function of gain and smoothing. In these sessions, a different set of gain and smoothing parameters were imposed for each four-minute block of data. During each block, participants acquired targets that appeared in an alternating fashion in either the center of the workspace or in one of eight radially spaced outer locations. We collected two additional center-out-back sessions specifically for this study that further explored the effect of smoothing on online performance, yielding a total of 15 sessions. Each session is listed in Supplemental Table 1.

We also used 9 random target sessions that were originally reported in^30^ to validate the PLM’s ability to predict performance as a function of target distance and radius. In the random target task, after a target was acquired a new target appeared in a random location within the square workspace with uniform probability (but was constrained to appear far enough away from the cursor so as not to overlap it). Targets appeared with a radius chosen from a set of 1 of 3 possible radii. Each session is listed in Supplemental Table 2.

Finally, to demonstrate the versatility of the PLM for helping to design new decoding innovations, we used it to design a static nonlinear function that transforms the speeds decoded by a linear velocity decoder. We collected four additional sessions (two with T5 and two with T8) that measured the online performance benefits of using the model-designed nonlinearity. Each session is listed in Supplemental Table 3.

### Session structure and task

To control the cursor, participants were instructed to attempt to make arm movements (T5, T8), imagine moving the thumb and index finger (T6), or imagine moving a computer mouse placed under the hand (T7); these different instructions were consistent with each participant’s preferred and successful prior strategies. Each session began with an open-loop block where participants watched the cursor automatically complete a center-out-and-back target acquisition task while imagining or attempting to make the cursor movements shown. We used this data to calibrate the decoding matrix. Then, participants completed a series of closed-loop neural control blocks with computer assistance that were used to re-calibrate the decoder. Finally, the decoder was held fixed and participants completed a series of 4 or 5 minute closed-loop blocks with no computer assistance. Data reported in the study are from these later closed-loop blocks.

Participants acquired targets by holding the cursor in unbroken contact with the target region for a specified dwell-time. A trial was failed and the cursor was reset to the target position if a maximum movement time was exceeded. After a target was acquired, another target appeared shortly afterwards (0–300 ms).

### Online decoding framework

The decoding methods used here are described in detail in^27,30^. Briefly, we used a linear velocity decoder that is a reparameterized version of the steady-state, velocity Kalman filter^19,22,24^. Our decoder parameterization explicitly separates the dimensionality reduction step (the mapping between the high dimensional neural activity to the two dimensional decoded velocity) from the smoothing dynamics and overall gain. This parameterization allowed us to isolate and control the decoder’s gain and smoothing properties while keeping its dimensionality reduction step constant throughout the session.

In the dimensionality reduction step, the neural features (threshold crossing rates and spectral power within the 250–5000 Hz band) were mapped to a decoded “control” vector at each time step with the equation

$${u}_{t}=D{f}_{t}$$

where *f*_*t*_ is an N × 1 neural feature vector, *D* is a 2 × N decoding matrix, and *u*_*t*_ is a 2 × 1 decoded control vector. The decoded control vector was then smoothed using the following dynamical equation that determines the cursor velocity

$${v}_{t+1}=\alpha {v}_{t}+(1-\alpha )\beta {u}_{t}$$

where *v*_*t*_ is cursor velocity, $\alpha \in [0,1)$ parameterizes the amount of smoothing and $\beta \in (0,\infty )$ parameterizes the gain. Note that the above parameterization can describe any decoder that is composed of linear dimensionality reduction plus exponential smoothing (e.g.^3,13,15,16^).

The *D* matrix was calibrated with optimal linear estimation (T8)^7,45^, reverse regression (T6, T7)^45^, or by estimating the Kalman gain matrix (T5)^19^. The user’s intention was estimated with the “unit vector” intention estimation method (T6, T7, T8) or ReFIT (T5)^23^. Importantly, we normalized the decoding matrix *D* so that β alone parameterizes the maximum speed of the cursor. When *D* is normalized, β defines the cursor’s “terminal velocity”, or the (average) speed that the cursor would asymptotically approach if the user pointed *u*_*t*_ in the same direction forever. We report cursor gain as this maximum speed, reported in units of target distances per second (TD/s).

### Piecewise-linear feedback control model

The piecewise-linear model (PLM) we used to simulate and predict online performance is described in detail in^27^ and is illustrated in Fig. 2. Here, we give a brief overview of the PLM. The PLM describes the decoded control vector (*u*_*t*_) at each time step as the sum of an intentional component (*c*_*t*_, the “encoded” control vector) and the decoding error (*e*_*t*_):

$${u}_{t}={c}_{t}+{e}_{t}$$

The encoded control vector represents neural modulation that drives the cursor towards the target. The model uses piecewise-linear functions to describe *c*_*t*_ as a function of the target position and the user’s internal estimate of cursor position and velocity:

$${c}_{t}=\frac{{g}_{t}-{\hat{p}}_{t}}{\Vert {g}_{t}-{\hat{p}}_{t}\Vert }{f}_{targ}(\Vert {g}_{t}-{\hat{p}}_{t}\Vert )+\frac{{\hat{v}}_{t}}{\Vert {\hat{v}}_{t}\Vert }{f}_{vel}(\Vert {\hat{v}}_{t}\Vert )$$

where *g*_*t*_ is the target position,${\hat{p}}_{t}$ is the user’s internal estimate of cursor position, ${\hat{v}}_{t}$ is the user’s internal estimate of cursor velocity, and *f*_*targ*_ and *f*_*vel*_ are piecewise-linear, one-dimensional weighting functions that are fit empirically to the data (the Model Fitting section below provides more details). Essentially, this equation models *c*_*t*_ as the sum of a point-at-target vector (weighted by the function *f*_*targ*_) and a velocity damping vector (weighted by *f*_*vel*_). Note that the simulated user does not have direct access to the true cursor position and velocity, but instead uses an “internal estimate” of cursor position and velocity (${\hat{{\rm{p}}}}_{{\rm{t}}}$ and ${\hat{{\rm{v}}}}_{{\rm{t}}}$) made from delayed visual feedback and a forward model matched to the decoder dynamics (motivated by the result in^46^).

The internal state estimates ${\hat{p}}_{t}$ and ${\hat{v}}_{t}$ are generated as follows. At each time step *t*, the user receives perfect knowledge of the delayed cursor state ${x}_{t-\tau }$, where $\tau$ is the user’s visual feedback delay (in # of time steps). After receiving delayed feedback, the user employs a forward model (matched perfectly to the cursor dynamics), combined with knowledge of previously issued control signals, to estimate the current cursor state *x*_*t*_ by running the decoder equations forward starting from ${x}_{t-\tau }$:

$${\hat{x}}_{t}={A}^{\tau }{x}_{t-\tau }+\sum _{i=0}^{\tau -1}{A}^{i}B{c}_{t-i-1},$$

where ${\hat{x}}_{t}$ is the user’s internal model estimate of the cursor state (${\hat{x}}_{t}$ is a vector containing ${\hat{p}}_{t}$ and ${\hat{v}}_{t}$). The A and B matrices parameterize the decoder dynamics in state space form^27^ $({x}_{t}=A{x}_{t-1}+B{u}_{t})$. Since *c*_*t*_ differs from *u*_*t*_ for all time steps from (t − τ − 1) to (t − 1), ${\hat{x}}_{t}$ will differ from *x*_*t*_. Essentially, this means that the user can only counteract decoding noise perturbations τ time steps after they occur, limiting the effectiveness of the user’s feedback corrections.

The decoding error *e*_*t*_ is characterized with an autoregressive noise model

$${e}_{t}={{\rm{\Pi }}}_{1}{e}_{t-1}+{{\rm{\Pi }}}_{2}{e}_{t-2}+\ldots +{{\rm{\Pi }}}_{p}{e}_{t-p}+{\varepsilon }_{t}$$

where Π_i_ are 2 × 2 matrices (for a 2D task), *p* is the number of time lags in the model, and *ε*_*t*_ is zero-mean, multivariate Gaussian i.i.d. noise. In addition to describing the noise magnitude, the autoregressive model describes the frequency content of the noise by parameterizing correlations and anti-correlations in time. The matrices Π_i_ and the covariance matrix of *ε*_*t*_ are fit empirically to the data. The number of time lags *p* is chosen during model fitting by increasing *p* until the ability to predict the decoding error on held-out data does not improve.

### Additions made to the model to increase predictive power

We made two changes to the model as originally described in^27^ in order to increase its predictive power on held-out data. First, we added signal-dependent noise. That is, we modeled how the variance of *ε*_*t*_ changed as a function of the magnitude of *c*_*t*_. To do so, we scaled the covariance matrix of *ε*_*t*_ at each time step by multiplying it by a weighting function ${f}_{SDN}(\Vert {c}_{t}\Vert )$ that we estimated empirically. We found that this improved prediction accuracy for T6, whose neural signals’ noise had a small but significant signal dependence; it didn’t affect prediction accuracy for T7 or T8, whose noise was almost entirely signal independent^30^.

The second change was to add a simple model of user adaptation. In the original study^27^, the model was only used to simulate movements under the same gain and smoothing condition to which it was fit. Here, since we used the model to predict how performance would change in *different* conditions, we needed to consider how user behavior (i.e. the *f*_*targ*_ and *f*_*vel*_ functions) might change under these different conditions. In the original study^27^, we found that users adapt their neural modulation to adjust for large gain or smoothing values that create significant second-order acceleration dynamics. To predict this adaptation, we ran an optimization routine to search for new *f*_*vel*_ functions that would lead to the highest simulated performance under the new gain and smoothing settings of interest. We assumed that *f*_*vel*_ would be linear (as found in^27^) and constrained our search by looking for the slope that would minimize average movement time. To search for the slope, we did a brute force search over a likely range of values (from 0 to −3 in steps of 0.1). This model of adaptation essentially assumes that the user will rapidly adapt their behavior to add the appropriate amount of velocity damping called for by the gain and smoothing settings. Note that this method requires no data from held-out conditions, since it only searches for the *f*_*vel*_ function that causes the simulated user to perform the best (as opposed to the *f*_*vel*_ function that leads to the highest match to held-out data).

### Model fitting

The PLM parameters are empirically fit to a set of observed cursor movements (*p*_*t*_, *v*_*t*_) and decoder outputs (*u*_*t*_). The control policy model (*f*_*targ*_ and *f*_*vel*_) and the user’s internal model estimates of the cursor state (${\hat{p}}_{t}$, ${\hat{v}}_{t}$) are fit together in an iterative process:

1. Initialize the internal model estimates to a delayed cursor state (${\hat{p}}_{t}={p}_{t-\tau }$, ${\hat{v}}_{t}={v}_{t-\tau }$).
2. Using the current internal model estimates, fit the control policy model parameters using least squares regression to minimize the error between the modeled control vector *c*_*t*_ and the observed decoder output *u*_*t*_.
3. Using the current control policy model, update the internal model estimates assuming that the user employs a forward model that is matched perfectly to the cursor dynamics (i.e., for each time step, begin with delayed cursor states and step forward using the cursor dynamics and efference copies of recent control vectors until the current time step).
4. Return to step 2 until we have completed five iterations.

Once the control policy model is fit, the noise model is then fit to characterize the error time series *e*_*t*_ = *u*_*t*_ - *c*_*t*_. To do so, first the Π_i_ matrices are fit using least squares to minimize the prediction error of *e*_*t*_. To choose the number of time lags to include in the autoregressive noise model, we started at zero and increased the number of lags by 1 until the cross-validated predictive power of the model stopped increasing. The noise covariance of ${\varepsilon }_{t}$ is then estimated from the covariance of the prediction errors of the autoregressive noise model. Finally, the signal dependent noise function ${f}_{SDN}(\Vert {c}_{t}\Vert )$ is found by estimating the signal-specific covariance matrix of *ε*_*t*_ for twenty different bins corresponding to different levels of $(\Vert {c}_{t}\Vert )$. The bin edges were evenly spaced from $\Vert {c}_{t}\Vert =0$ to $\Vert {c}_{t}\Vert =1.5$. Scalar values of ${f}_{SDN}(\Vert {c}_{t}\Vert )$ for each bin were found by estimating the bin-specific covariance matrix of the noise and then finding the scale factor to best relate it to the full-data covariance matrix using a least squares fit.

### Model prediction

To test the PLM’s ability to predict online performance, its performance was evaluated on held-out data only. That is, the model parameters were fit using data from one condition and then used to predict performance under a different condition. To predict performance as a function of gain and smoothing, we fit the model to one block of data per session and then used that model to predict online performance for all other blocks in that session. For participants T7 and T8, we chose to fit the model to the block with the lowest gain setting, reasoning that in a practical use case calibration data would most likely be collected at a low gain setting. For participant T6, this approach did not always work as well when predicting performance under high gain conditions. We believe this is because low gain blocks do not always contain many examples of near-target corrective behavior and corresponding decoding noise, which may be necessary to accurately predict this behavior in high-gain conditions for some participants. Therefore, for predicting the performance of T6, we fit the noise model on the block with the highest gain in any given session. However, this necessitated that we fit the control policy model (*f*_*targ*_) using a slow speed decoder calibration block so that T6’s maximum neural modulation could still be reliably estimated (for very high gains, users decrease the magnitude of their neural modulation to adapt^27^). Note that, although the fitting procedure was not the same as the other participants, no held-out data of any kind was used to inform the model for T6. Also, reasonably high performance across all participants could still be obtained regardless of which blocks were used to fit the model (Supplemental Fig. 2).

To predict performance as a function of target radius and distance, we fit the model to a subset of trials recorded on each day during a random target task. This subset of trials included data only from far away targets (target distance in the top 25% of all trials) and small target radii (the smallest of 3 radii tested). The PLM was then used to predict performance for all other target radii and distances on that day. The model fitting procedure was the same for both participants T6 and T8.

### Design of a nonlinear speed transform

We collected two sessions each of data with T5 and T8 comparing the performance of a standard decoder to one with a model-optimized nonlinear speed transform. T8 completed a standard center-out-back 2D cursor control task. T5 completed a center-out-back 4D cursor control task where he was required to move a 3-dimensional bar to the location of a target bar and to rotate the bar to match the orientation of the target. T5 continuously controlled the bar’s velocity in the X, Y, and Z dimensions and the rate of change of a single rotational degree of freedom. The rotational degree of freedom was not treated any differently by the decoder and behaved the same way as the translational degrees of freedom. In these tasks, targets were evenly spaced along a circle (T8, 8 targets) or a 4D hypersphere (T5, 80 targets).

We optimized the speed transform separately for each session. First, we fit the PLM to an initial block of data collected at the beginning of the session. We then optimized gain, smoothing, and the nonlinear transform parameters to minimize total movement time on a difficult target acquisition task. T8 completed a task with a 4 second dwell time and where the effective target radius (target plus cursor radius) was equal to 1/8^th^ of the target distance. T5 completed a task with a 1 second dwell time and an effective target radius of 1/10^th^ the target distance. A trial was considered failed if a maximum movement time of 10 seconds (T5) or 12 seconds (T8) was exceeded.

For T8, the nonlinear transform was parameterized as a piecewise linear function with 14 breakpoints. First, the optimal gain and smoothing parameters were found for a standard linear decoder using an exhaustive search. Then, the breakpoints for the nonlinear transform were spaced evenly from 0 to 1.25*β (where β is the optimized gain). An iterative search was performed to find the optimal function values at these breakpoints using MATLAB’s “patternsearch” function that implements a direct search method. The objective function was defined as the average movement time of 200 simulated movements. We performed 24 searches and then averaged the 24 resulting functions to yield the final result. For T5, the nonlinear transform was parameterized more compactly as an exponentiation: ${s}_{out}=\,{{s}_{in}}^{p}$, where *s*_*out*_ is the output speed, *s*_*in*_ is the input speed, and *p* is an exponentiation parameter. We optimized over gain, smoothing and *p* simultaneously using an exhaustive grid search.

Once the nonlinear transform was optimized, we then collected a series of alternating five-minute blocks where T5 and T8 used a decoder with model-optimized gain and smoothing values either with or without the nonlinearity. Across both days for each participant, we collected 5 blocks using the standard decoder and 6 blocks using the nonlinear transform with T5, and 9 blocks of each with T8. To make Fig. 8, we pooled the data across all of these blocks.

## Supplementary information

Supplementary Information

## Data Availability

The data can be made available upon reasonable request by contacting the lead or senior authors.
