# Supplementary material for: Principled BCI Decoder Design and Parameter Selection Using a Feedback Control Model
Source: Sci Rep. 2019 Jun 20;9:8881. doi: 10.1038/s41598-019-44166-7 (PMC6586941; doi:10.1038/s41598-019-44166-7)
Supplement: Supplementary file 1 — Supplementary Information [file 41598_2019_44166_MOESM1_ESM.pdf]

Supplementary Materials for "Principled BCI Decoder Design and Parameter Selection Using a Feedback Control Model"

Francis R. Willett, Daniel R. Young, Brian A. Murphy, William D. Memberg, Christine H. Blabe, Chethan Pandarinath, Sergey D. Stavisky, Paymon Rezaii, Jad Saab, Benjamin L. Walter, Jennifer A. Sweet, Jonathan P. Miller, Jaimie M. Henderson, Krishna V. Shenoy, John D. Simeral, Beata Jarosiewicz, Leigh R. Hochberg, Robert F. Kirsch, A. Bolu Ajiboye

***Section 1: Simulation Methods for Figure 1*..2**

***Section 2: Additional Simulations for Figure 1*..3**

***Section 3: Sensitivity of the Model to Training Data*..4**

***Section 4: Dataset Details*..5**

***Section 5: Within-session Predictions*..8**

***Section 6: Joint Optimization of Gain, Smoothing and an Exponential Speed Transform*.........12**

Section 1: Simulation Methods for Figure 1

The simulation shown in Figure 1 in the main text is meant to give a simple demonstration of how standard decoder calibration methods, which are based on minimizing offline prediction error, can fail to yield decoder dynamics that maximize online performance.

For all simulated blocks, we simulated 40 neural features with Gaussian noise that were linearly tuned to a linear control policy at each time step. That is, the 40 *x* 1 neural feature vector f_t at each time step *t* was given by the equation

$$f_t = E(g_t - p_t) + \varepsilon_t,$$

where *E* is a 40 x 2 matrix of tuning coefficients that determined the preferred direction and depth of modulation of each feature, g_t is a 2 x 1 target position vector, p_t is a 2 x 1 cursor position vector, and $\varepsilon_t \sim N(0, \Sigma)$ is a 40 x 1 Gaussian noise vector. The preferred directions contained *E* were uniformly distributed and their depth of modulation was set equal to 1. The covariance matrix Σ was a diagonal matrix with all diagonal entries equal to 2. The target position, g_t, was a distance of 1 away from the center of the workspace.

While we could have used the PLM to simulate a more realistic control policy, neural noise and visual feedback delay, we chose this simple model instead to show that these extra factors are not necessary for causing an offline vs. online performance discrepancy.

The simulated blocks were generated as follows. First, we simulated an open-loop dataset that consisted of 80 minimum-jerk [2], center-out trajectories that lasted 750 ms each. A velocity Kalman filter was calibrated using this dataset according to the methods in Gilja et al. 2012 [3]. Then, this initial decoder was used in closed-loop to generate the first closed-loop block of center-out data ("OL Cal Block" in Fig. 1) consisting of 40 movements. We then proceeded to simulate a series of closed-loop blocks, with each one using a decoder that was calibrated on data from the previous closed-loop block ("ReCal 1-5" in Fig. 1) with 40 movements each. In the "small target" task, the target radius was equal to 1/16; in the "large target" task, the target radius was equal to 4/16. The dwell time was 1 second in all tasks. To estimate the user's intended velocity during both the open-loop and closed-loop blocks, we started with the original velocity vectors and then rotated them to point towards the target and zeroed them when the cursor was overlapping the target (following the ReFIT calibration method [3]). To generate the performance surfaces, 40 movements were simulated for each parameter pair.

In Supplemental Figure 1, we show that a failure to optimize online performance is not due to a mismatch between the user's control policy and the assumptions made by ReFIT; even if $g_t - p_t$ is taken as the user's "intended velocity", the decoder is still suboptimal.

[1] F. R. Willett *et al.*, "Feedback control policies employed by people using intracortical brain–computer interfaces," *J. Neural Eng.*, vol. 14, no. 1, p. 016001, Feb. 2017.

[2] T. Flash and N. Hogan, "The coordination of arm movements: an experimentally confirmed mathematical model," *J. Neurosci.*, vol. 5, no. 7, pp. 1688–1703, 1985.

[3] V. Gilja *et al.*, "A high-performance neural prosthesis enabled by control algorithm design," *Nat. Neurosci.*, vol. 15, no. 12, pp. 1752–1757, Dec. 2012.

Section 2: Additional Simulations for Figure 1

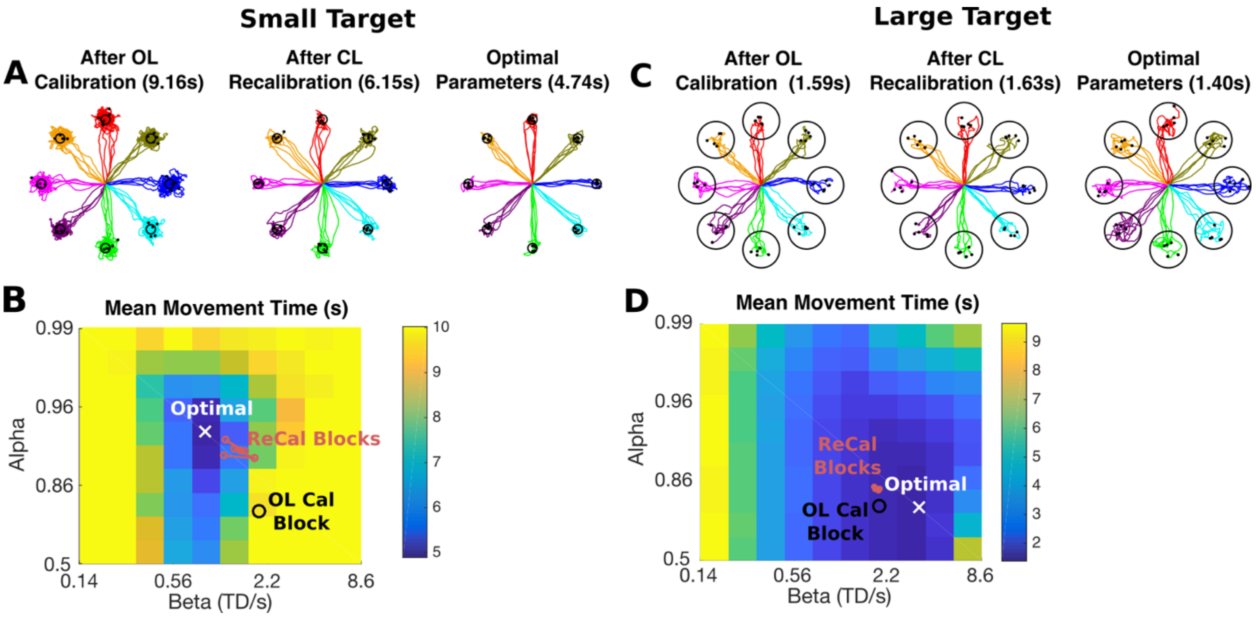

Supplemental Figure 1. Identical to Figure 1 in the main text, except that the user's true intent is used to calibrate the decoder (see Supplemental Section 2 for more details). The results demonstrate that a failure to optimize online performance is not due to a mismatch between the user's control policy and the assumptions made by ReFIT. Even though there is a perfect match between what the user's neural activity is encoding and what is assumed during decoder calibration, the decoder takes on suboptimal gain and smoothing parameters (even when allowed to recalibrate). Here, unlike in the case of ReFIT, continued recalibration does not change the parameters very much (they appear to jitter around a fixed point that is suboptimal).

Section 3: Sensitivity of the Model to Training Data

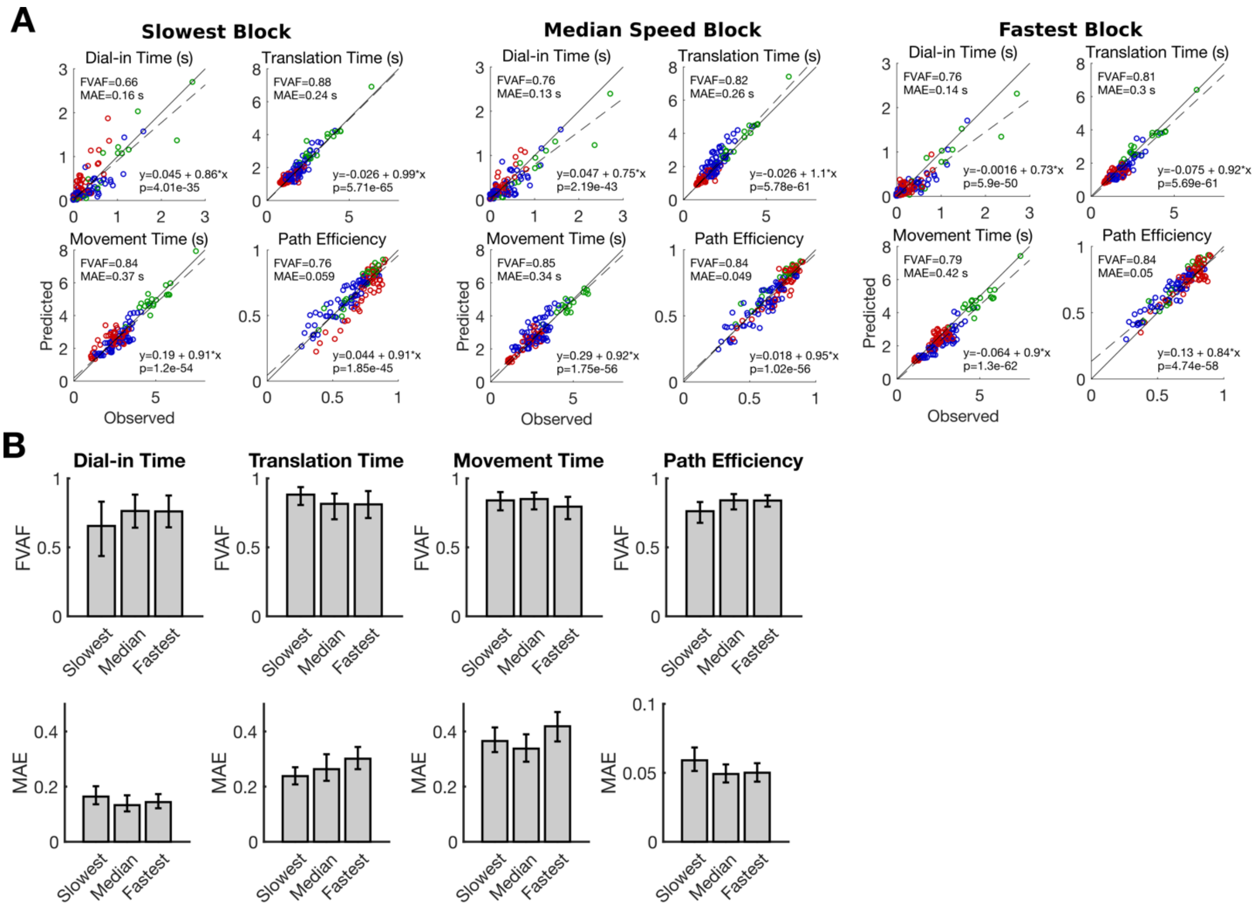

Supplemental Figure 2. To assess the PLM's sensitivity to the type of block it was trained on, we replicated the summary analyses from Figure 5 for three different training conditions: either the PLM was fit to the lowest gain block for each session, the median gain block, or the highest gain block. This tests the PLM's performance when trained under three very different regimes of cursor movement dynamics. (A) The analysis in Figure 5B is replicated for each type of block. Overall, good quality predictions are achieved for each of the three fitting conditions. Each circle on each panel represents the average performance for one block. In the top left corner of each panel, the fraction of variance accounted for by the model's predictions (FVAF) is shown in addition to the mean absolute error of the predictions (MAE). To assess the model's bias and statistical significance, a linear regression was performed for each panel that regressed the model's predictions against the observed data. The regression coefficients are shown in the bottom right corner and indicate low bias (the slopes are near one and the intercepts are near zero). The regression line is plotted as a dashed black line and the unity line as a solid black line for comparison. Finally, the p-value for the slope coefficient is reported. (B) The FVAF (fraction of variance accounted for) and MAE (mean absolute error) are summarized for each metric; error bars indicate 95% confidence intervals (computed by bootstrap resampling the blocks that were included). No large differences in performance are apparent.

Section 4: Dataset Details

Participant	Session Type	Date (& Post-Implant Day)	# of Blocks	Dwell Time (s)	Target Distance / (Target + Cursor Diameter)	Max Movement Time	Task Visualization	Motor Cue
T6	Mixed Gain/Smooth	2014.11.05 (699)	2	1	3.27	10	Cursor	Imagined Index + Thumb
T6	Mixed Gain /Smooth	2014.11.10 (704)	8	1	3.27	10	Cursor	Imagined Index + Thumb
T6	Mixed Gain /Smooth	2014.11.19 (713)	8	1	3.27	10	Cursor	Imagined Index + Thumb
T6	Gain	2014.12.10 (734)	8	0.15	3.27	10	Cursor	Imagined Index + Thumb
T6	Gain	2015.01.14 (769)	8	0.15	3.27	10	Cursor	Imagined Index + Thumb
T6	Smoothing	2015.01.21 (776)	8	1	3.27	10	Cursor	Imagined Index + Thumb
T6	Smoothing	2015.02.02 (788)	8	1	3.27	10	Cursor	Imagined Index + Thumb
T7	Mixed Gain /Smooth	2014.09.08 (406)	10	0.5 or 1	3.27	8.5 or 10.5	Cursor	Attempted Mouse
T7	Mixed Gain /Smooth	2014.09.11 (409)	10	1	3.27	10.5	Cursor	Attempted Mouse
T8	Mixed Gain /Smooth	2015.01.26 (57)	7	0.5	1.96	8	Cursor + Arm	Attempted Arm
T8	Mixed Gain/Smooth	2015.01.27 (58)	10	0.5	1.96	8	Cursor + Arm	Attempted Arm
T8	Gain	2015.03.24 (114)	15	0.15	1.96	8	Cursor + Arm	Attempted Arm
T8	Smoothing	2015.06.30 (212)	12	0.5	1.96	8	Cursor + Arm	Attempted Arm
T8	Smoothing	2016.12.22 (387)	8	0.5	1.96	8	Cursor + Arm	Attempted Arm
T8	Smoothing	2016.02.01 (428)	10	0.5	1.96	8	Cursor + Arm	Attempted Arm

Supplemental Table 1. A list of all gain and smoothing sessions included in the study.

Participant	Date (& Post-Implant Day)	Cursor Gains (WW/s)	# of blocks	Cursor Smoothing Alpha	Dwell Times (s)	Effective Target Radii (WW)	Task Visualization	Motor Cue
T6	2015.03.06 (820)	0.52, 1.04	5 per gain	0.92	0.75	0.07, 0.10, 0.13	Cursor	Imagined Index + Thumb
T6	2015.03.16 (830)	1.09	10	0.92	0.75	0.07, 0.10, 0.13	Cursor	Imagined Index + Thumb
T6	2015.03.23 (837)	3.06	6	0.92	0.15	0.07, 0.10, 0.13	Cursor	Imagined Index + Thumb
T8	2015.03.11 (101)	0.43	15	0.96	0.5	0.11, 0.14, 0.17	Cursor + Arm	Attempted Arm
T8	2015.03.17 (107)	0.74	16	0.94	0.75	0.10, 0.12, 0.16	Cursor + Arm	Attempted Arm
T8	2015.05.12 (163)	1.2	8	0.94	0.75	0.10, 0.12, 0.16	Cursor + Arm	Attempted Arm
T8	2015.05.28 (179)	0.57	4	0.94	0.75	0.10, 0.12, 0.16	Cursor + Arm	Attempted Arm
T8	2015.08.31 (274)	0.40, 0.64	7 per gain	0.94	0.75	0.10, 0.12, 0.16	Cursor + Arm	Attempted Arm
T8	2015.11.19 (354)	0.26	8	0.96	0.75	0.10, 0.12, 0.16	Cursor + Arm	Attempted Arm

Supplemental Table 2. A list of all random target sessions included in the study. Cursor gains and effective target radii are reported using a unit of distance equal to the width of the square workspace (WW or “workspace width”). Effective target radii are equal to the cursor radius plus the target radius and define the region where the center of the cursor must dwell to acquire the target.

Participant	Date (& Post-Implant Day)	# of Blocks	Dwell Time (s)	Target Distance / (Target + Cursor Diameter)	Max Movement Time	Task Visualization	Motor Cue
T8	2016.06.29 (577)	10	4	4	12	Cursor + Arm	Attempted Arm
T8	2016.07.06 (584)	8	4	4	12	Cursor + Arm	Attempted Arm
T5	2017.09.25 (404)	5	1	5	10	4D Cursor	Attempted Arm
T5	2017.10.04 (413)	6	1	5	10	4D Cursor	Attempted Arm

Supplemental Table 3. Parameters for the four sessions that measured the benefit of using a nonlinear speed transform function.

Section 5: Within-session Predictions

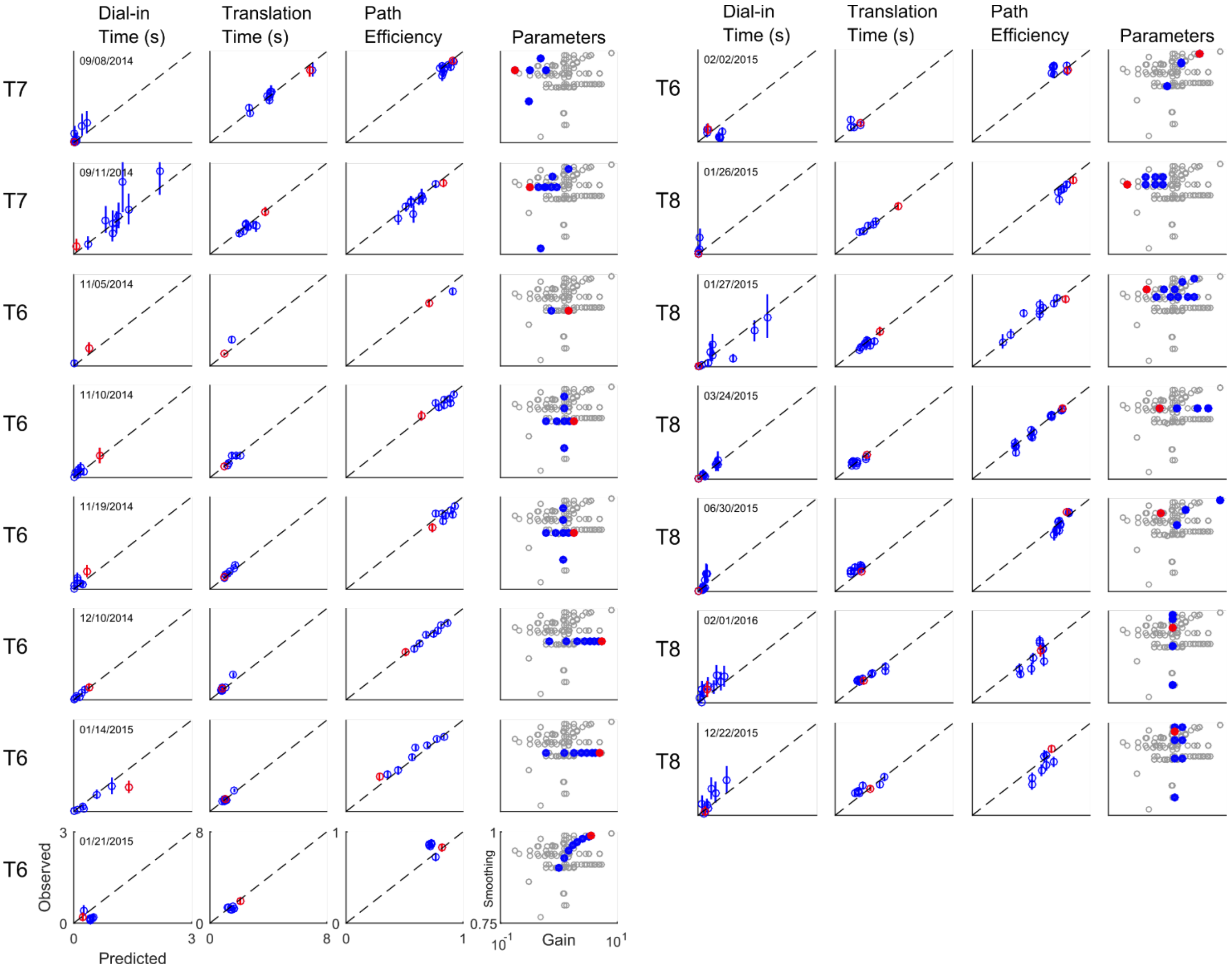

Supplemental Figure 3. The data shown in Figure 3 is broken apart by individual session, where each four-panel row shows data from a single session. Red circles indicate blocks on which the PLM was fit. The results indicate that the PLM is successful at accounting for the within-session variability in performance caused by the gain and smoothing parameters alone. The across-session variability in performance is caused not only by different gain and smoothing parameters, but also by different levels of decoding noise occurring on different days and by each participant's different overall levels of decoding noise, different feedback delays, and/or different control policies.

An alternative version of this figure with zoomed-in axes for each panel is provided below in Supplemental Figure 4.

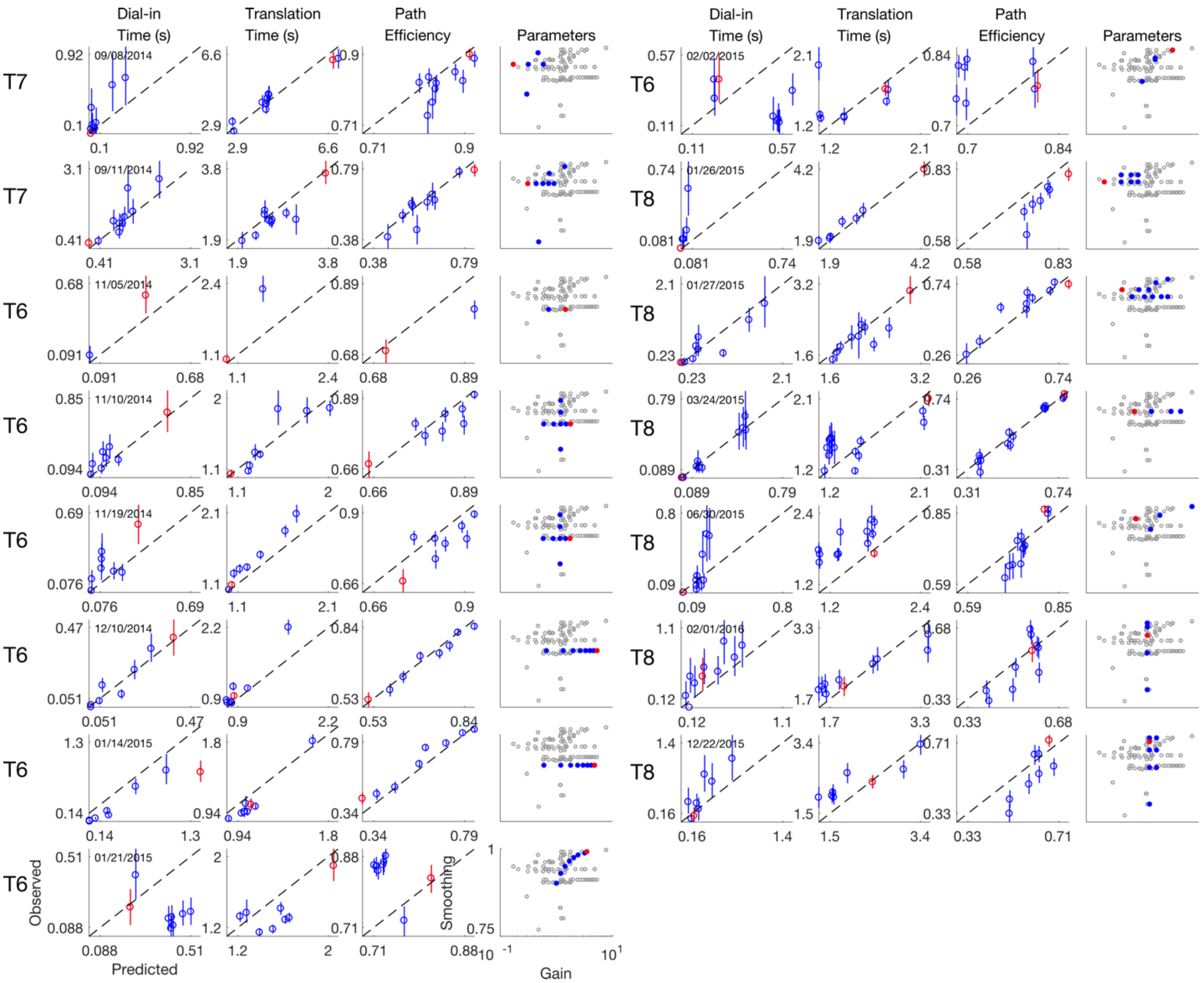

Supplemental Figure 4. An alternative version of Supplemental Figure 3 with zoomed-in axes for each panel to show more detail (but note that each panel has different axes).

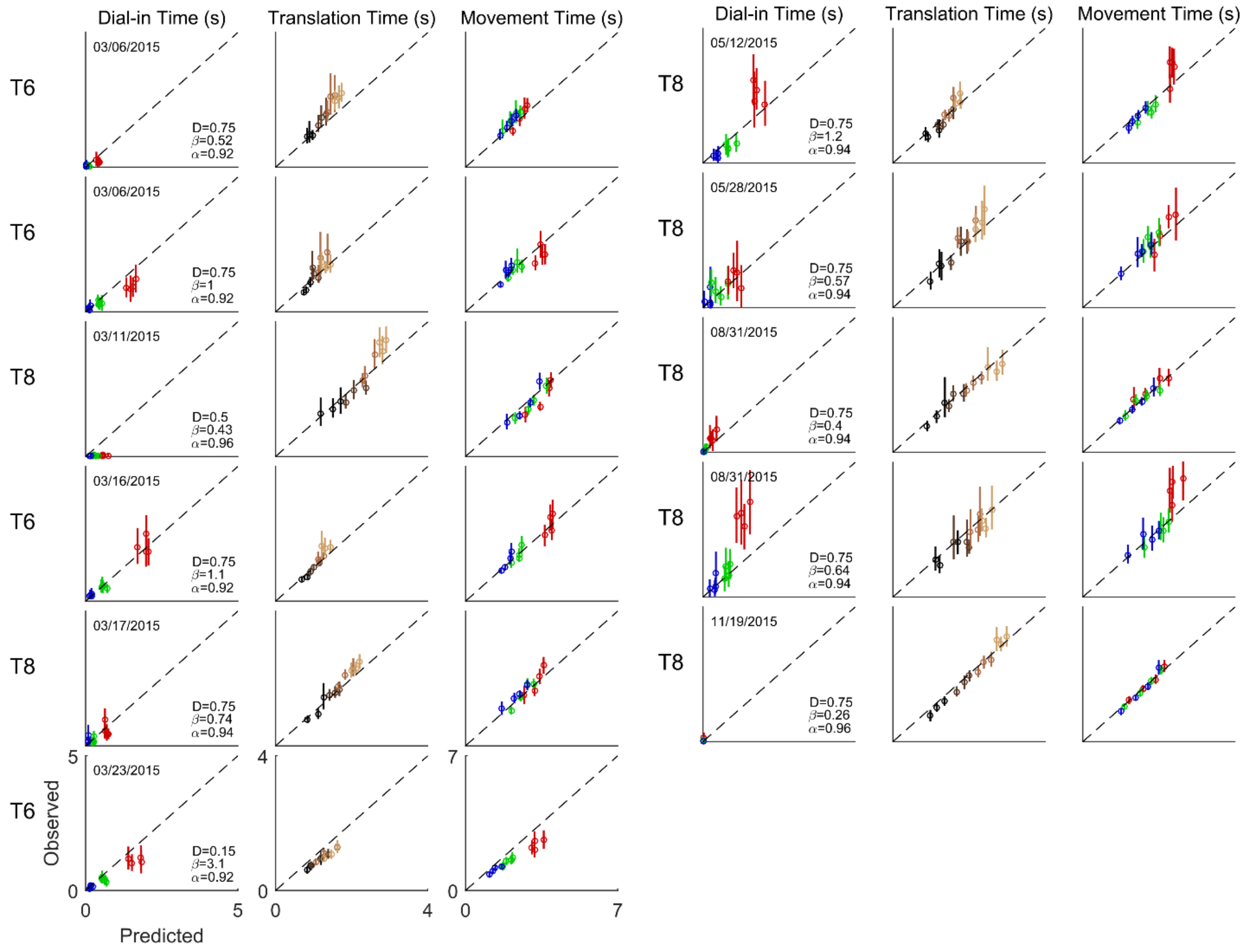

Supplemental Figure 5. The ability of the PLM to predict how online performance will change as a function of target radius and distance, broken apart by each random target session and each gain and smoothing setting tested within that session. Each three-panel row plots data from a single session and gain/smoothing setting. Data from each target radius and target distance pair is plotted as a single circle (3 radii and 4 distances make for 12 pairs per panel).

For the dial-in time and movement time panels, the circles are colored by target radius (red = small, green = medium, blue = large). For the translation time panel, the circles are colored by target distance (dark = close, light = far). For each row, the dwell time (D), gain (β), and smoothing setting (α) are indicated.

An alternative version of this figure with zoomed-in axes for each panel is provided below in Supplemental Figure 6.

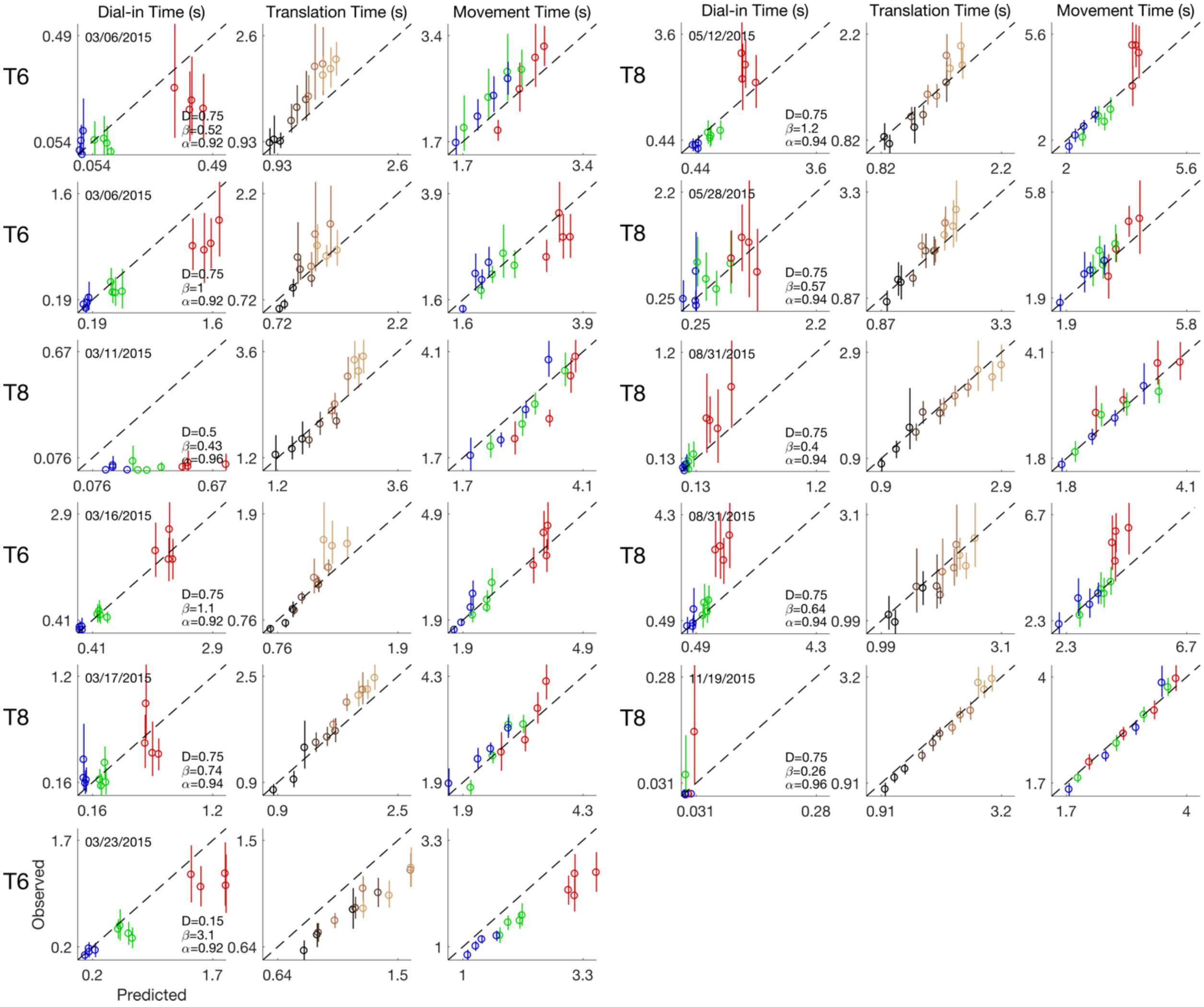

Supplemental Figure 6. An alternative version of Supplemental Figure 5 with zoomed-in axes for each panel to show more detail (but note that each panel has different axes).

Section 6: Joint Optimization of Gain, Smoothing and an Exponential Speed Transform

Here we argue that adding an exponential nonlinearity by itself, without optimizing its parameters in concert with decoder gain and smoothing, my not lead to any performance improvement. Thus, although the idea of an exponential nonlinearity could have been easily conceived without a model like the PLM, there would be no straightforward way to optimize it or accurately measure its performance improvement relative to an optimized linear decoder. We illustrate this point with a simulation experiment.

For this experiment, we first fit the PLM parameters to a block of T8 data. Then, we simulated performance when using an exponential nonlinearity for a difficult task with a dwell time of 2 seconds and an effective target radius (target plus cursor radius) equal to 1/10 of the target distance. We performed separate gain and smoothing parameter optimizations for each exponent value (16 different exponent values evenly spaced from 1 to 4). The results (Supplemental Figure 7) show that there is an optimal exponent at 2.6 that decreases the total movement time from 5.8 seconds to 4.8 seconds. Importantly, the optimal gain and smoothing values depend on the exponent, indicating that this is a difficult joint optimization problem. We can't necessarily expect good performance simply by taking good gain and smoothing values for a linear decoder (exponent = 1) and then simply increasing the exponent. Panel B illustrates this point explicitly. In panel B, simulated movement time is shown when taking the optimal gain and smoothing values for a linear decoder (exponent = 1) and then increasing the exponent. Performance only improves slightly for small exponents (<1.2) and at 2.6 actually decreases performance substantially.

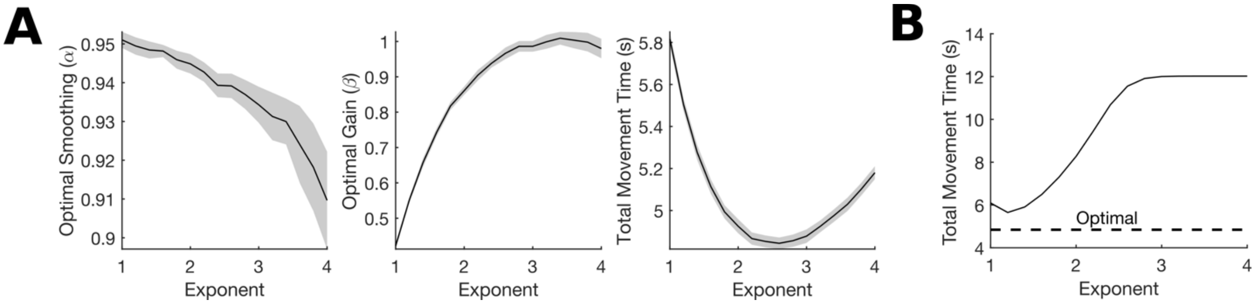

Supplemental Figure 7. Optimal gain and smoothing parameters change as a function of the exponent of an exponential speed transform function (as used in Fig. 8E-H). (A) A separate gain and smoothing optimization was completed for each exponent value; as the exponent increases, the optimal gain increases and the optimal smoothing value decreases. The optimal gain and smoothing values for the best exponent (2.6) are quite different from those of a linear decoder (exponent = 1). Shaded regions indicate 95% confidence intervals (30 separate optimizations were performed). (B) Combining an exponential nonlinearity with gain and smoothing values that were optimal for the linear case (exponent = 1) does not lead to the same performance improvement. In fact, performance is worse when the exponent is 2.6.

Why does the optimal gain increase as a function of the exponent? The exponential nonlinearity causes speeds less than 1 to become slower; thus, the gain must be increased to compensate. But how much should the gain increase? This question is difficult to answer in a quantitatively precise way without using a model.

It may be helpful to consider what it might have taken to perform the experiment in Figure 8 without the PLM. First, gain and smoothing values would have had to been swept through trial and error to find

good values for the linear decoder. Then, for several different candidate exponents, more gain and smoothing parameter sweeps would have had to have been performed. This could easily consume weeks of valuable experimental time. Moreover, the results may not have been accurate, since decoding noise can vary from day to day, making optimal parameters on one day potentially suboptimal on another day. Finally, how could we confirm that the experimenter had swept enough values to ensure a fair comparison? In many studies, these factors are not considered and performance for only a single set of decoder parameters are reported, making it unclear how the results would change if the parameters were changed. The PLM provides a fast and objective way to jointly optimize over several parameters at the same time, making for more objective and informative decoder comparisons.
